# A Multimodal Approach to Measuring Listening Effort: A Systematic Review on the Effects of Auditory Task Demand on Physiological Measures and Their Relationship

**DOI:** 10.1097/AUD.0000000000001508

**Published:** 2024-06-17

**Authors:** Laura Keur-Huizinga, Sophia E. Kramer, Eco J. C. de Geus, Adriana A. Zekveld

**Affiliations:** 1 Amsterdam UMC location Vrije Universiteit Amsterdam, Otolaryngology—Head and Neck Surgery, Ear & Hearing, Amsterdam Public Health Research Institute, Amsterdam, The Netherlands; 2 Department of Biological Psychology, Vrije Universiteit Amsterdam, Amsterdam, The Netherlands.

**Keywords:** Brain activity, Listening effort, Mental effort, Psychophysiology, Speech perception

## Abstract

**Objectives:**

Listening effort involves the mental effort required to perceive an auditory stimulus, for example in noisy environments. Prolonged increased listening effort, for example due to impaired hearing ability, may increase risk of health complications. It is therefore important to identify valid and sensitive measures of listening effort. Physiological measures have been shown to be sensitive to auditory task demand manipulations and are considered to reflect changes in listening effort. Such measures include pupil dilation, alpha power, skin conductance level, and heart rate variability. The aim of the current systematic review was to provide an overview of studies to listening effort that used multiple physiological measures. The two main questions were: (1) what is the effect of changes in auditory task demand on simultaneously acquired physiological measures from various modalities? and (2) what is the relationship between the responses in these physiological measures?

**Design:**

Following Preferred Reporting Items for Systematic Reviews and Meta-Analyses (PRISMA) guidelines, relevant articles were sought in PubMed, PsycInfo, and Web of Science and by examining the references of included articles. Search iterations with different combinations of psychophysiological measures were performed in conjunction with listening effort-related search terms. Quality was assessed using the Appraisal Tool for Cross-Sectional Studies.

**Results:**

A total of 297 articles were identified from three databases, of which 27 were included. One additional article was identified from reference lists. Of the total 28 included articles, 16 included an analysis regarding the relationship between the physiological measures. The overall quality of the included studies was reasonable.

**Conclusions:**

The included studies showed that most of the physiological measures either show no effect to auditory task demand manipulations or a consistent effect in the expected direction. For example, pupil dilation increased, pre-ejection period decreased, and skin conductance level increased with increasing auditory task demand. Most of the relationships between the responses of these physiological measures were nonsignificant or weak. The physiological measures varied in their sensitivity to auditory task demand manipulations. One of the identified knowledge gaps was that the included studies mostly used tasks with high-performance levels, resulting in an underrepresentation of the physiological changes at lower performance levels. This makes it difficult to capture how the physiological responses behave across the full psychometric curve. Our results support the Framework for Understanding Effortful Listening and the need for a multimodal approach to listening effort. We furthermore discuss focus points for future studies.

## INTRODUCTION

Since 2011, research related to hearing and audiology has increasingly focused on the application of multiple physiological measures as markers of effortful listening (Mackersie & Cones 2011). Mental effort is defined as “the deliberate allocation of mental resources to overcome obstacles in goal pursuit when carrying out a task” and listening effort as “a specific form of mental effort that occurs when a task involves listening” (Pichora-Fuller et al. 2016). When listening becomes more difficult, for example, due to loud background noise or impaired hearing ability, more mental resources and effort are required to perceive the relevant auditory stimuli, such as speech (Wingfield et al. 1999; Piquado et al. 2012). Individuals who are hard-of-hearing may need to allocate increased effort to function normally in everyday life due to their hearing loss and/or due to unwanted background sounds (Alhanbali et al. 2017). If such increased effort is expended frequently and over a long time, it can lead to higher levels of fatigue and stress. This in turn increases the risk of developing physical and/or mental complications, such as cardiovascular disease and depression (Lin et al. 2008; Pronk et al. 2011; Pichora-Fuller et al. 2015). In addition, hearing loss can lead to decreased social participation and isolation, also lowering quality of life (Mick et al. 2014). Reducing listening effort in daily life may aid rehabilitation or prevent such complications. It is therefore important to better understand the complexity of effortful listening and critically assess which physiological measures reliably reflect listening effort.

There are many factors that affect listening effort such as task difficulty (Mackersie & Cones 2011; Bertoli & Bodmer 2016; Ohlenforst et al. 2018), available mental resources (Pichora-Fuller 2003; Wingfield et al. 2006), and motivation of the person to perform well (Richter 2016; Koelewijn et al. 2018). A common strategy to manipulate listening effort is to vary the auditory task demand by changing characteristics of the auditory signals. Examples of changes to the target signal include vocoded or accented speech. In addition, the ratio between the sound levels of simultaneously presented target speech and background noise can be adapted (signal to noise ratio [SNR]). Listening effort generally shows a non-monotonic relationship with task demand: first, effort will increase with increasing task demand, but the person will be inclined to invest less effort when the task becomes very hard, and ultimately give up when it becomes impossible (Kahneman 1973; Yerkes & Dodson 1908; Pichora-Fuller et al. 2016; Kuchinsky & Vaden 2020). It is important to note that this relationship is influenced by many factors, such as mental resources, fatigue, motivation, reward, task context, or the presence of another individual (Richter 2010; Pichora-Fuller et al. 2016; Ohlenforst et al. 2018; Plain et al. 2020; Pielage et al. 2021). The Framework for Understanding Effortful Listening (FUEL) illustrates how effort varies as a function of demand and motivation (Pichora-Fuller et al. 2016; Figure 2, pp. 16S), where effort is highest when both motivation and listening demand are high (but not impossible). In addition, the motivational intensity theory (Brehm & Self 1989; Richter et al. 2016) on the mobilization of effort in goal pursuit explains how various factors moderate the relationship between effort and task demand. For example, when motivation and success importance are high, the amount of effort a person is willing to invest will be higher or more sustained. When the task is too difficult or not worth the effort, the exerted effort will drop. As opposed to this saw tooth shape, Kuchinsky and Vaden (2020) proposed an inverted-U shaped function, where listening effort gradually declines after maximum effort is reached. The exact shape of the relationship between demand and effort is unknown, and will therefore be referred to as non-monotonic for the purpose of this review.

Changes in auditory task demand, by manipulating characteristics of the auditory signals, can influence physiological measures that are widely assumed to reflect the resulting changes in listening effort (Pichora-Fuller et al. 2016; Peelle 2018; Strand et al. 2020). Such physiological measures include cardiovascular measures (heart rate variability [HRV], blood pressure), pupillometry, and brain activity (electroencephalography, functional magnetic resonance imaging) (for overviews, see e.g., Pichora-Fuller et al. 2016; Peelle 2018; Zekveld et al. 2018; Richter et al. 2023). Later, we will summarize relevant physiological measures that have been applied to assess the effect of auditory task demand on listening effort (Table 1).

**TABLE 1.** Physiological measures and the expected direction of effect with increased effort

Technique	Measure	Physiology	Unit	Expected Direction of Effect With Increased Effort
Cardiovascular (electro-/impedance cardiography)	Heart rate	ANS	bpm	↑
	Heart rate variability	PNS	msec	↓
	Pre-ejection period	SNS	msec	↓
Electrodermal activity	Skin conductance level	SNS	µS	↑
	Skin conductance responses	SNS	ppm	↑
Pupillometry	Pupil dilation	ANS	mm	↑
Endocrine factors	Cortisol	NES	nmol/L	↑
	Chromogranin A	NES	ng/mL	↑
	Alpha-amylase	NES	U/mL	↑
Electroencephalography	Alpha power	CNS	μV	↑/↓
	Beta power	CNS	μV	↑/↓
	Delta power	CNS	μV	↑
	Theta power	CNS	μV	↑
	Event-related potential response: P3 and N100	CNS	µV	↑
	Event-related potential response: N400	CNS	µV	↓
Functional magnetic resonance imaging	Blood oxygenation level-dependent response	CNS	A.U.s	↑
Functional near-infrared spectroscopy	Cerebral oxygenation	CNS	µmol	↑
Electromyography	Facial EMG activity	SoNS	μV	↑

ANS, autonomic nervous system; A.U.s, arbitrary units; bpm, beats per minute; CNS, central nervous system; µS, micro Siemens; µV, micro volt; NES, neuroendocrine system; PNS, parasympathetic nervous system; ppm, peaks per minute; SNS, sympathetic nervous system; SoNS, somatic nervous system.

### Physiological Measures

Physiological measures include those reflecting changes in, for example, the autonomic, somatic, and central nervous systems (CNS) and the neuroendocrine system. The somatic nervous system is part of the peripheral nervous system involved in muscular activities. Measures from the CNS mostly involve cortical brain activity. The neuroendocrine system includes the set of organs or cells involved with hormone secretion, such as the hypothalamus and pituitary gland. The autonomic nervous system (ANS) is the part of the peripheral nervous system that regulates involuntary physiological activity and can be divided in the sympathetic, parasympathetic, and enteric branches. For this review, only the sympathetic and parasympathetic branches of the ANS were considered. Increases in sympathetic nervous system (SNS) activity are associated with the “fight or flight” response and increases in activity of the parasympathetic nervous system (PNS) with “rest and digest” (McCorry 2007; Gibbons 2019). These responses predominantly refer to physical activity, for which activity of the two branches is often reciprocal: SNS activity increases and PNS activity decreases with increased physical activity and vice versa. However, under conditions of mental load and psychological stress, more complex patterns can appear next to the classical reciprocal activation. Provided the person is motivated to perform well (Richter & Gendolla 2009; Seery 2011; Richter 2016), increased demands of mentally challenging tasks cause changes in SNS and PNS activity that may follow the reciprocal pattern: sympathetic outflow to the organs is increased and PNS activity is decreased (Venables & Fairclough 2009). The relevant physiological measures will be discussed in more detail.

### Measures of Combined PNS and SNS Activity

Measures reflecting a mixture of both SNS and PNS activity include heart rate (HR) and pupil dilation. HR is the number of heart beats per minute, but can also be reflected by the average inter-beat-intervals (IBI) per minute, both as derived from the electrocardiogram (ECG). Increased SNS and decreased PNS activity result in an increase in HR and a decrease in IBI, and vice versa. Generally, HR is expected to increase with increasing effort. HR has been shown to slightly increase with effortful listening (Seeman & Sims 2015). However, some studies have found no effect of effortful listening on HR (Plain et al. 2021; Slade et al. 2021).

Changes in task-induced pupil dilation can reflect the amount of effort that is put into completing that task, given that other factors such as luminance are kept constant (Beatty & Kahneman 1966; Kahneman & Beatty 1966; Winn et al. 2018). Pupil dilation is also sensitive to changes in the participant’s attention and engagement in the task, as well as to the emotional content of stimuli (Kret et al. 2013). The pupil response is the change in pupil diameter evoked by a stimulus, calculated relative to a baseline pupil size determined prior stimulus presentation (Winn et al. 2018). Pupil dilation is one of the most widely used physiological markers of listening effort (for an overview, see Zekveld et al. 2018) and follows the hypothesized non-monotonic relationship with task demand levels (Ohlenforst et al. 2017; Wendt et al. 2018). Specific measures of task-induced pupil dilation in the time-domain include the (absolute) pupil size, mean pupil dilation (MPD), and peak pupil dilation (PPD). The PPD is the maximum pupil dilation compared with baseline within a specified time interval. Furthermore, analysis on the pupil time course can be performed using principal component analysis and growth curve analysis. Principal component analysis divides the pupil response into separate components and extracts features from each component (Verney et al. 2004), whereas growth curve analysis assesses change over time using multilevel polynomial regression (Mirman et al. 2008). Pupil dilation reflects an unknown and multidimensional mixture of activity in both PNS and SNS circuits, influenced by complex patterns of activity in several neuromodulatory systems (Joshi & Gold 2020; Strauch et al. 2022). An important route by which the CNS affects pupil dilation is the norepinephrine system of the locus coeruleus. Increased activity of this system has been associated with cognitive performance and pupil size changes (Joshi et al. 2016; Strauch et al. 2022). However, note that most models of the neural underpinnings of the pupillary response attribute the cognition-related responses and other complex attention-related processes (Zekveld et al. 2018; Joshi & Gold 2020; Strauch et al. 2022). This makes it unlikely that the pupillary response simply reflects a general measure of effort but rather one important aspect of the complex effort response.

In an attempt to disentangle the contribution of the PNS and SNS to the pupil response, ambient light manipulations have been used (Steinhauer & Hakerem 1992). In darkness, the pupillary sphincter muscles are relaxed, so the influence of PNS inhibitory activity on the pupil size is considered negligible (Loewenfeld & Lowenstein 1993). Steinhauer et al. (2004) suggested that the pupil dilation response to an arithmetic task was predominantly mediated by inhibition of PNS activity at the oculomotor nucleus. In addition, Wang et al. (2018) showed smaller pupil responses in light for hard-of-hearing subjects compared with normal-hearing listeners, but similar responses in dark, suggesting reduced PNS inhibition for the hard-of-hearing subjects.

#### Measures Sensitive to SNS Activity

Two measures that exclusively reflect SNS activity include pre-ejection period (PEP) and electrodermal activity (EDA). PEP reflects SNS effects on cardiac contractility, and is not influenced by PNS activity (Harris et al. 1967; Cacioppo et al. 1994; Behnke & Kaczmarek 2018). PEP can be derived from the combination of ECG and impedance cardiogram, which is a measure of resistance against an alternating electrical current across the thorax. PEP represents the time between the electrical systole (Q-wave on the ECG signal) and the opening of the aortic valve (B-point on the impedance cardiogram signal) (Mendes 2009). An increase in cardiac SNS activity results in a decrease in PEP, which shortens as contractility increases. So, as SNS activity is expected to increase with listening effort, PEP is expected to decrease. In a few studies, PEP has been shown to follow a non-monotonic relationship with task demand in the expected direction (Richter et al. 2008; Mallat et al. 2020; Slade et al. 2021).

EDA is the electrical conductivity of the skin and represents the amount of sweat that is produced by eccrine glands, which are innervated exclusively by the sympathetic branch of the ANS (Lidberg & Wallin 1981; Dawson et al. 2017). SCL is a tonic measure of sweat gland activity and fluctuations in SCL reflect general changes in alertness, but can also be influenced by unintended effects from environmental temperature changes (Boucsein 2012; Dawson et al. 2017). In addition, skin conductance responses (SCRs) are rapid phasic changes in the low-pass filtered SCL signal, generally with an amplitude of at least 0.01 microSiemens (Braithwaite et al. 2013). These rapid SCL increases can be induced by task-relevant or emotionally salient external stimuli. An increase in SCL and SCRs reflect an increase in SNS activity (Boucsein 2012). Consequently, EDA measures are expected to increase with increasing listening effort, which has been confirmed in multiple studies (Mackersie & Cones 2011; Francis et al. 2016; Cvijanović et al. 2017). However, it is important to note that due to the aforementioned unintended effects and inter-individual differences, it is difficult to quantify a meaningful change in SCL (for details, see e.g., Boucsein 2012; Braithwaite et al. 2013).

#### Measures Sensitive to PNS Activity

A widely used measure that reflects PNS activity is HRV, which is the beat-to-beat variability in IBIs over a certain period of time. The variability is mainly affected by respiration (Grossman & Kollai 1993; Eckberg 2003) and slow blood pressure oscillations (de Boer et al. 1985; Legramante et al. 1999). HRV due to blood pressure fluctuations reflects both PNS and SNS activity, but HRV associated with cardiorespiratory coupling, also known as respiratory sinus arrhythmia (RSA), largely reflects PNS activity. This is because SNS signaling at the heart, in contrast to PNS signaling from the vagus nerve, is not fast enough to affect changes in HR on a breath-to-breath level in the typical frequency band of respiration (Chapleau & Sabharwal 2011; de Geus et al. 2019). RSA can be obtained through time- or frequency-domain methods (Massaro & Pecchia 2016). The peak-to-valley method is one example in the time-domain and calculates the mean difference between the longest IBI during expiration and the shortest IBI during inspiration for each respiratory cycle. Another time-domain measure, that does not require co-registration of respiration, is the root mean square of successive differences (RMSSD). In the frequency domain, the high frequency (HF) HRV is obtained from the spectral density spectrum of the equidistant resampled IBI times series, most often computed with discrete Fourier transformation. Note that measures such as the low-frequency power and the low-frequency/HF ratio in the frequency domain are also considered HRV, but not exclusively indicative of PNS activity. For the purpose of this review, we will refer to HRV as HRV measures of the PNS (RSA, HF-HRV, RMSSD). As PNS activity is expected to decrease with increasing listening effort, (pv)RSA, RMSSD, and HF-HRV are all expected to decrease. Although some studies did show a decrease of these HRV measures during effortful listening (Seeman & Sims 2015; Mackersie & Calderon-Moultrie 2016; Mackersie & Kearney 2017), several others have not found any effects (Cvijanović et al. 2017; Plain et al. 2021; Slade et al. 2021).

### Neuroendocrine System

Two important neuroendocrine systems that are involved in the stress response are the hypothalamic pituitary adrenal axis and the sympathetic adrenal medullary axis. Acute stress induces elevated hypothalamic pituitary adrenal activity and leads to an increase in cortisol secretion from the adrenal cortex (Kirschbaum & Hellhammer 1989). Similarly, increased sympathetic adrenal medullary reactivity is followed by an increase in adrenaline from the adrenal medulla. In addition, sympathetic activity increases alpha-amylase (Nater et al. 2006) and chromogranin A (CgA) secretion (Kanamaru et al. 2006; Den et al. 2011). These three endocrine factors can be obtained through saliva. Secretion of these endocrine factors is expected to increase with increasing effort. Currently, only two studies have used endocrine factors as biomarker for listening effort, but these did not yield promising results (Kramer et al. 2016; Zekveld et al. 2019).

### Somatic Nervous System

Muscle tension is a measure of the somatic nervous system and tends to increase with effort, which can be detected through electromyography by surface electrodes placed over a muscle (Van Boxtel & Jessurun 1993). Facial electromyography (fEMG) measures the tension of the face muscles and is widely used to investigate emotional expressions and valence (Larsen et al. 2003). In particular, the corrugator and frontalis muscles are sensitive to fatigue (Veldhuizen et al. 2003) and mental effort (Van Boxtel & Jessurun 1993; Waterink & van Boxtel 1994). Facial EMG activity is expected to increase with increasing listening effort, as shown by Mackersie and Cones (2011) in the frontalis muscle. However, Francis et al. (2021) did not replicate this effect for the corrugator supercilii.

### Central Nervous System

Listening effort can also be assessed by investigating the effect of auditory task demand manipulation on cortical brain activity. A frequently used electroencephalography (EEG) measure is alpha power, that is, the oscillations in the alpha frequency band (8 to 13 Hz). Alpha power in the parietal cortical areas has been studied most frequently in the context of listening effort as it is associated with relevant factors such as attention (Klimesch 2012), working memory (Jensen et al. 2002), and inhibition of task-irrelevant information (Strauß et al. 2014). Inconsistent results have been reported: for example, Obleser and Weisz (2012) found increased alpha power with decreasing intelligibility, whereas Miles et al. (2017) found the opposite pattern of results. In addition, frontal theta power (4 to 8 Hz) is associated with working memory during effortful listening (Wisniewski et al. 2018), delta power (1 to 3 Hz) has been associated with the processing of linguistic properties and the comprehension of speech (Etard & Reichenbach 2019), and beta power (16 to 30 Hz) has been associated with sentence processing (Lam et al. 2016). Theta and delta power increase with increasing listening effort (Wisniewski et al. 2015; Corcoran et al. 2022), but no association has been found for beta power (Dimitrijevic et al. 2019; Corcoran et al. 2022). Furthermore, the event-related potential response has been associated with several functions related to auditory and language processing, such as auditory attention (Lewald et al. 2018) and semantic memory access (Aydelott et al. 2006). For example, the P3 and N100 amplitude components increase with auditory task demand (Obleser & Kotz 2011; Bertoli & Bodmer 2014; Lewald et al. 2018), whereas N400 amplitude decreases (Obleser & Kotz 2011; Silcox & Payne 2021). Last, neural tracking of speech (the cross-correlation of EEG and the temporal envelope of speech) is sensitive to speech intelligibility (Muller et al. 2019). In addition, poorer hearing acuity is associated with a smaller difference in neural tracking between interfering speech and target speech, negatively affecting the ability to filter the interfering speech signal (Petersen et al. 2017).

Functional magnetic resonance imaging (fMRI) is commonly used to investigate task-evoked changes in brain activity. It measures the blood oxygenation level-dependent response: when neuronal activity increases, blood vessels provide an excess supply of oxygenated blood resulting in changes in the relative concentration of oxygenated hemoglobin. This induces variations in the magnetic field around the blood vessels (Ogawa et al. 1990, 1993; Lindquist et al. 2009). Similarly, functional near-infrared spectroscopy (fNIRS) measures the concentration of hemoglobin in the blood vessels in extracerebral and cerebral brain tissues using near-infrared light. An advantage of fNIRS over fMRI is that it is much quieter and not limited by any magnetic items or implants of patients. In contrast, verbally answering during a task is not possible, as it disturbs the measurement of task-evoked responses. fMRI studies have observed increased activation with increasing listening effort in the bilateral superior temporal gyrus (Zekveld & Kramer 2014) and frontal brain regions (Wild et al. 2012). Using fNIRS, the activation in the left inferior frontal cortex was found to peak around 25 to 50% intelligibility level (Lawrence et al. 2018), reflecting the expected non-monotonic relationship between listening effort and auditory task demand.

### Current Review

Previous research on listening effort has used a wide variety of physiological measures, but these do not allow a uniform “best measure” for listening effort to be defined (Strand et al. 2020; Richter et al. 2023). It is also unclear how these different physiological measures relate to each other and whether they all demonstrate a comparable non-monotonic relationship with auditory task demand. An added complexity is that there is a lot of variety in how auditory task demand was manipulated in the listening tasks and what type of stimuli was used (such as sentences, words, or clips). In this review, we deliberately focused on studies simultaneously assessing multiple physiological measures during effortful listening tasks. This provides the opportunity to test the relationships between the different measures. To the authors’ knowledge, a systematic review including studies on listening effort exclusively using multiple physiological measures has not been performed thus far.

The aim of the current study was to perform a systematic review of the extant research using multiple physiological measures during auditory tasks. We had two main questions: (1) what is the effect of changes in auditory task demand on simultaneously acquired physiological measures? and (2) what is the relationship between the responses in these physiological measures? In addition, in the included articles, the relationship between physiological and subjective measures was also explored, if applicable. With this review, we aimed to shed light on the current understanding of the physiological measurements of listening effort and highlight the current knowledge gaps and necessary focus points for future studies.

## MATERIALS AND METHODS

The current systematic review was performed according to the Preferred Reporting Items for Systematic Reviews and Meta-Analyses (PRISMA) guidelines (Page et al. 2021). English peer-reviewed articles were identified by using relevant search terms (see Appendix in Supplemental Digital Content 1, http://links.lww.com/EANDH/B381, which lists the search terms) in PubMed, PsycInfo, and Web of Science and by examining the references of included articles. Final identification was performed in May 2023. Articles were included if they investigated the effect of auditory task demand manipulations on changes in at least two different physiological (including neuroimaging) measures in adult humans, either normal hearing and/or hard-of-hearing. Case reports or reviews were excluded. Papers were excluded if the participants were children (<18 years old) or if only one physiological measure was applied. Articles were also excluded if the main focus of the study was not to investigate whether the physiological measures (dependent variables) were sensitive to changes in auditory task demand (independent variable). In addition, articles were excluded if they were published before the year 2000, as the field using physiological parameters as a measure of listening effort is relatively young. A review protocol was not prepared before identification.

The physiological measures in the search query included cardiovascular measures (HR, blood pressure, PEP, HRV), EDA (SCL, SCR), endocrine measures (cortisol, alpha-amylase, CgA), pupillometry-based measures (pupil dilation), muscle tension (fEMG), and brain activity (EEG, magnetoencephalography, fMRI, fNIRS) (see Appendix in Supplemental Digital Content 1, http://links.lww.com/EANDH/B381). To limit the output in the databases to research articles including at least two measures, iterations of all different combinations were performed in conjunction with listening effort-related search terms. Articles were first selected based on the title and abstract. A final inclusion decision was made based on the full text. The main selection of articles was done by one reviewer. If they were unsure about the inclusion of an article, independent evaluation of the articles was performed by two other reviewers, and the inclusion of the article was discussed until consensus was reached.

For each included article, the main characteristics of the (in)dependent variables and participants were summarized in a table by one reviewer. This included the sample size and details of sex and age, as well as hearing status (normal hearing or hard-of-hearing). In addition, the psychophysiological measures of interest and task manipulations were listed. Lastly, relevant outcomes were listed: the performance levels on the tasks (if applicable), the effect of auditory task demand manipulations on the physiological measures, the correlations between these measures, and the test statistics, *p* values, and effect sizes. If details were missing, it was stated or not included in the table.

To assess the quality of the included articles, the Appraisal tool for Cross-Sectional Studies was used (Downes et al. 2016). It provides a guideline to rate the quality of studies and assess the risk of bias across multiple disciplines. The tool consists of 20 yes/no questions. The included articles were independently assessed by two reviewers. Any discrepancies were discussed afterward until consensus was reached.

Although subjective measurements were not the focus of this review, these will be briefly discussed as well. These include (rating) questions following a condition or task, for example regarding the perceived difficulty or effort. Although subjective measurements are weakly correlated with physiological measures (Shields et al. 2023), they are nevertheless relevant as they provide a measure of the perceived exerted effort or difficulty of the task. We discussed the results of subjective measurement tools if applied in the included articles and their relationships with the physiological measures.

## RESULTS

**Fig. 1.**
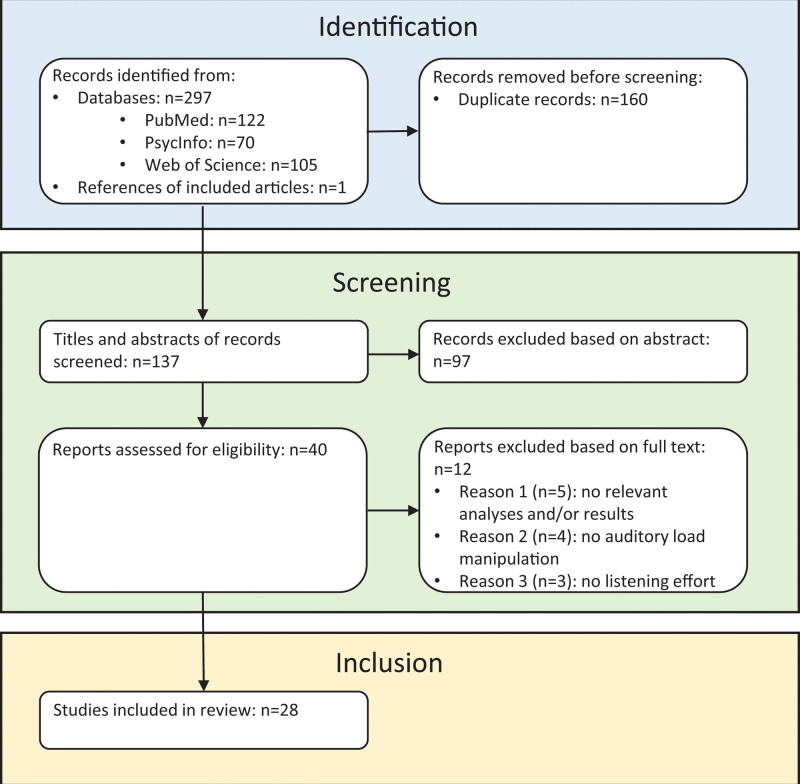
Preferred Reporting Items for Systematic Reviews and Meta-Analyses (PRISMA) flowchart of assessment process for identification, screening, and inclusion of records.

The query in the three databases identified 297 articles (Fig. 1): 122 articles in PubMed, 105 articles in Web of Science, and 70 articles in PsycInfo. In addition, one article was retrieved from reference lists of included articles. Before selection, duplicate articles were removed, which resulted in the omission of 160 articles. On the basis of title and abstract, full texts of 40 articles were checked for eligibility, of which fifteen were evaluated by the two other reviewers independently in addition to the first reviewer. As a result, 28 articles were included. Twelve articles were excluded: four studies did not have (relevant) auditory task demand manipulations, five did not have relevant research questions and analyses, and three did not have relevant methods. Supplementary Table 1 (see Table in Supplemental Digital Content 2, http://links.lww.com/EANDH/B382, which provides a detailed overview of the included articles) summarizes the relevant methods and results from the included articles in detail, in alphabetical order of the first author.

In the included studies, auditory task demand was manipulated using different methods and tasks. Most studies used sentences presented in background noise (13) and/or distorted sentences (6), such as vocoded speech. Other studies used audio clips in noise (four) or digits in noise (two). Remaining methods that were only used once were words in noise, communication between participant pairs in noise, tone frequency discrimination, digit sequences from a single or multiple talkers, dichotic digit task, and diotic-dichotic digits task. The most frequently applied tasks involved repetition of the target stimulus or answering comprehension questions about its content. Auditory task demand was mostly manipulated by varying the SNR, type of background noise, stimulus complexity/structure, speech rate, or the level of distortion. Details for each study can be found in Supplementary Table 1 (see Table in Supplemental Digital Content 2, http://links.lww.com/EANDH/B382, which provides a detailed overview of the included articles). Furthermore, Table 2 shows an overview of the performance levels on the task. Six studies used an adaptive procedure such that the performance level was fixed for each participant (as independent variable). Three studies did not report (relevant) performance levels and were therefore not included in the table. Most of the included studies used tasks with a performance level of 70% or higher.

**TABLE 2.**
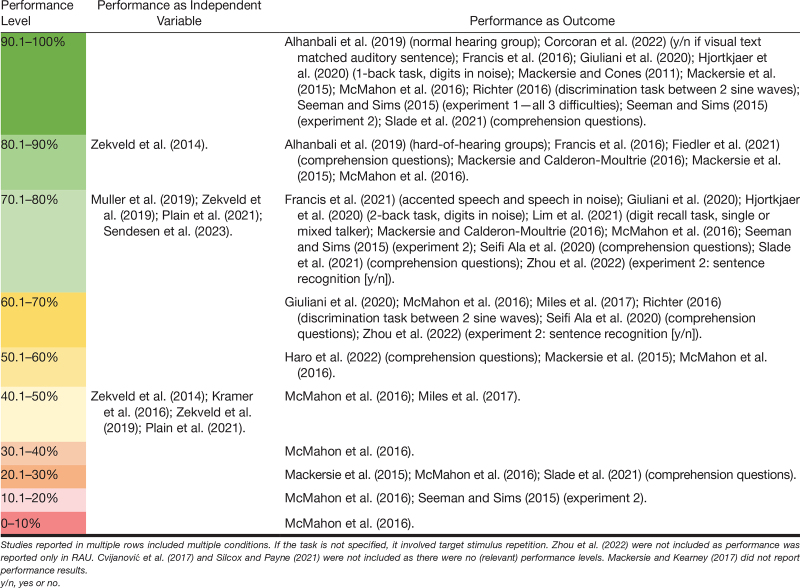
Performance levels as independent variable or outcome in included articles

Although changes in auditory task demand are often related to changes in task performance, please note that this was not the case for several studies. For example, Fiedler et al. (2021) did not find a main effect of hearing aid noise reduction or SNR (+3 dB versus +8 dB SNR) on performance, but there was an unintentional effect of talker, where performance was higher for the female talker compared with a male talker. Haro et al. (2022) found a main effect of condition on performance, but none of the post-hoc tests were significant. Seeman and Sims (2015) and Corcoran et al. (2022) also did not find any difference in performance between conditions. However, the subjective ratings in both studies indicated a difference in perceived difficulty between the conditions. Mackersie and Cones (2011), Mackersie and Calderon-Moultrie (2016), Hjortkjaer et al. (2020), and Francis et al. (2021) intentionally matched performance between conditions.

Pupillometry has been used most in the included studies (17 studies), next to EEG (12) and EDA (10). Of the EEG studies, alpha power was the most frequently investigated measure (nine). The combination of measures most frequently used among the articles was EEG with pupillometry (12 articles). Other combinations included EDA and HRV (five articles), EDA and HR (four), HRV and HR (three), PEP and HR (three), EDA and EMG (two), EDA and pupillometry (two), EMG and HR (two), pupillometry and endocrine factors (two), pupillometry and fMRI (one), pupillometry and fNIRS (one), HRV and PEP (one), and EDA and EEG (one).

### Effect of Auditory Task Demand

Table 3 provides an overview of the physiological responses to increases in auditory task demand. In case the study task included multiple conditions, this study was included in one of the effect columns if there was a significant effect for at least one condition (either a significant difference from baseline or between two conditions). Sendesen et al. (2023) and the SCL results of Alhanbali et al. (2019) were not included in the table due to missing formal analyses between the task (consisting of one condition) and baseline. In general, if an effect of auditory task demand on the physiological measure was found, the direction of this effect was consistent between studies. However, this was not the case for EEG alpha power, which has been shown to increase as well as decrease with increasing auditory task demand (Table 3).

**TABLE 3.** Overview of directions of effect with increasing auditory task demand per physiological measure

		Affected by Increasing Auditory Task Demand?		
	Measure	Increase in Outcome Measure	No Effect on Outcome Measure	Decrease in Outcome Measure
Cardiovascular (ECG/ICG)	HR	Francis et al. (2021); Richter (2016); Seeman and Sims (2015) (experiment 1)*; Seeman and Sims (2015) (experiment 2).	Francis et al. (2016)*; Mackersie and Cones (2011)*; Plain et al. (2021); Slade et al. (2021).	
	HRV†		Cvijanović et al. (2017); Mackersie et al. (2015) (for NH); Plain et al. (2021); Slade et al. (2021).	Mackersie and Calderon-Moultrie (2016)*; Mackersie and Kearney (2017); Mackersie et al. (2015) (for HH); Seeman and Sims (2015) (experiment 1)*; Seeman and Sims (2015) (experiment 2).
	PEP‡		Plain et al. (2021).	Richter (2016); Slade et al. (2021).
Electrodermal activity	SCL	Cvijanović et al. (2017); Francis et al. (2021); Mackersie and Calderon-Moultrie (2016)*; Mackersie and Cones (2011)*; Mackersie and Kearney (2017); Seeman and Sims (2015) (experiment 1; raw SCL)*.	Seeman and Sims (2015) (experiment 1: reactivity)*; Seeman and Sims (2015) (experiment 2).	
	SCR amplitude	Francis et al. (2016)*; Giuliani et al. (2020) (response window).	Giuliani et al. (2020) (speech presentation); Mackersie et al. (2015).	
	SCR frequency	Francis et al. (2021).	Francis et al. (2016); Cvijanović et al. (2017).	
Pupillometry	Pupil dilation/size	Alhanbali et al. (2019)§; Fiedler et al. (2021) (noise reduction*; Δtalker); Giuliani et al. (2020); Haro et al. (2022)*¶; McMahon et al. (2016) (cubic relationship); Miles et al. (2017); Muller et al. (2019); Seifi Ala et al. (2020); Silcox and Payne (2021); Zekveld et al. (2019).	Fiedler et al. (2021)* (ΔSNR); Hjortkjaer et al. (2020)*; Lim et al. (2021).	
	Peak pupil dilation	Zekveld et al. (2014); Kramer et al. (2016); Zekveld et al. (2019); Zhou et al. (2022).	Hjortkjaer et al. (2020)*.	
NES	Cortisol		Kramer et al. (2016).	Zekveld et al. (2019).
	CgA		Kramer et al. (2016).	
	Alpha-amylase	Zekveld et al. (2019).		
EEG	Alpha power	Alhanbali et al. (2019)§; McMahon et al. (2016) (ΔSNR, 16-channel vocoding).	Corcoran et al. (2022)*∥; Fiedler et al. (2021)* (ΔSNR and noise reduction); Hjortkjaer et al. (2020)*; Lim et al. (2021) (encoding); McMahon et al. (2016) (ΔSNR, 6-channel vocoding).	Fiedler et al. (2021) (Δtalker); Haro et al. (2022)*¶; Lim et al. (2021) (memory retention); Miles et al. (2017); Seifi Ala et al. (2020).
	Beta power		Corcoran et al. (2022)*∥.	
	Delta power	Corcoran et al. (2022)*∥.		
	Theta power	Corcoran et al. (2022)*∥.	Hjortkjaer et al. (2020)*; Seifi Ala et al. (2020).	
	N400 ERP			Silcox and Payne (2021).
	P3 ERP	Lim et al. (2021).		
	Neural tracking			Muller et al. (2019).
fMRI	BOLD response	Zekveld et al. (2014) (degradation).	Zekveld et al. (2014) (intelligibility).	
fNIRS	Cerebral oxygenation			Zhou et al. (2022).
EMG	Facial EMG activity	Mackersie and Cones (2011)*.	Francis et al. (2021).	

* Performance was not sensitive to task demand manipulations.

† HRV decreases when PNS activity decreases, that is, when effort and/or stress increase.

‡ PEP decreases when SNS activity increases, that is, when effort and/or stress increase.

§ Compared with baseline only.

¶ Auditory task demand was not manipulated, but auditory attention was.

∥ Difficulty was determined post-hoc based on the clarity ratings of noise-vocoded vs. sine-wave synthesized sentences.

BOLD, blood oxygen level dependent; CgA, chromogranin A; EEG, electroencephalography; ERP, event-related potential; fMRI, functional magnetic resonance imaging; fNIRS, functional near-infrared spectroscopy; HH, hard-of-hearing; HR, heart rate; HRV, heart rate variability; ICG, impedance cardiogram; NES, neuroendocrine system; NH, normal hearing; PEP, pre-ejection period; SCL, skin conductance level; SCR, skin conductance response; SNR, signal-to-noise ratio.

### Relationship Between Physiological Measures

Of the 28 included articles, 16 included analyses of the relationship between two different physiological outcome measures. The results are listed in Supplementary Table 1 (see Table in Supplemental Digital Content 2, http://links.lww.com/EANDH/B382, which provides a detailed overview of included articles) and summarized in Table 4, which provides an overview of results. In Table 4, all associations are between reactivity scores, except for Alhanbali et al. (2019). The relationships between measures can be classified into three types: (1) a relationship between the measures averaged over all conditions (Zekveld et al. 2014; Mackersie et al. 2015; Miles et al. 2017; Fiedler et al. 2021; Francis et al. 2021; Plain et al. 2021; Zhou et al. 2022); (2) a relationship tested separately for each condition (Francis et al. 2016; Kramer et al. 2016; McMahon et al. 2016; Miles et al. 2017; Alhanbali et al. 2019; Zekveld et al. 2019; Plain et al. 2021; Silcox & Payne 2021); (3) a relationship between the differences in the measures between conditions (Muller et al. 2019; Seifi Ala et al. 2020; Lim et al. 2021). The latter category is informative about how the changes between conditions in each of the measures are related to each other. This analysis may be less sensitive to individual differences in how the task manipulations translate to changes in each of the measures. Specifically, physiological measures depend on characteristics like age, BMI, fitness level, health, personality (Stemmler & Wacker 2010; Johannes & Gaillard 2014). In general, these individual differences were partially accounted for by applying a baseline correction. Such baselines relate to periods of resting, watching a neutral video, or very easy task conditions (e.g., listening to speech in quiet). Another important distinction to make is whether the association analyses were performed on inter-individual or intra-individual level. Two studies (McMahon et al. 2016; Miles et al. 2017) reported averaging the individual correlations between measures, whereas all other (14) studies assessed the correlations at the inter-individual level.

**TABLE 4.** Matrix of relationships between physiological measures reported in the included studies

		Cardiovascular (ECG/ICG)				EDA			Pupillometry		NES			EEG				fMRI	fNIRS
		BVPA	HRV (RMSSD)	HRV (HF)	PEP	SCL	SCR Ampl	SCR Freq	MPD	PPD	Cortisol	CgA	Alpha-Amylase	Alpha Power	N400 ERP	P3 ERP	Neural Tracking	BOLD	Oxygenation
Cardiovascular	HR		*r* = 0.26, *r* = −0.02, *r* = 0.01, *r* = 0.12, *r* = 0.10^1AB^	*r* = −0.07, *r* = −0.26, *r* = 0.19, *r* = −0.18, *r* = −0.08^1AB^	*r* = −0.16, *r* = 0.19, ***r* = −0.42**, ***r* = −0.32**, *r* = −0.19^1AB^		N.S.^2B^	N.S.^2B^											
	BVPA	—				*r* = −0.05^3A^		*r* = 0.04^3A^											
	HRV (RMSSD)	—	—	***r* =** **0.33**, *r* = −0.06, *r* = 0.06, *r* = −0.10, *r* = 0.06^1AB^	*r* = −0.16, *r* = −0.27, *r* = 0.16, *r* = 0.12, *r* = −0.04^1AB^														
	HRV (HF)	—	—	—	*r* = 0.08, ***r* = −0.43**, *r* = −0.27, *r* = −0.27, *r* = −0.27^1AB^	***r* = −0.41** ^4A^													
	PEP	—	—	—	—														
EDA	SCL	—	—	—	—	—		***r* = 0.30** ^3A^	*r* = 0.17^5B^	***r* = 0.21** ^5B^				*r* = 0.01, *r* = −0.044, ***r* = −0.25**^5B^					
	SCR ampl	—	—	—	—	—	—	N.S.^2B^											
	SCR freq	—	—	—	—	—	—	—											
Pupillometry	MPD	—	—	—	—	—	—	—	—					*r* = −0.08, *r* = 0.11, *r* = −0.15^5B^; *β* = −0.005, SE = 0.004 and *β* = –2.8, SE = 2.45^6A^; *r* = 0.05, *r* = −0.27 ^7C^; M *r* = −0.10, M *r* = 0.05^8B^; *r* = 0.02, *r* = 0.08, *r* = −0.04, *r* = −0.01, M *r* = 0.01^9AB^; *r* = −0.17^10C^	SSE: **0.69**, **−1.27**, 0.30, SSCDE: **1.40**, 0.39, −**1.01**^11B^	*r* = 0.07, *r* = −0.24^7C^			
	PPD	—	—	—	—	—	—	—	—	—	*r* = 0.04, *r* = 0.09, *r* = −0.06, *r* = −0.01, *r* = 0.13, *r* = 0.02^12B^		*r* = −0.15; *r* = −0.11; *r* = −0.18; ***r* = −0.26**; *r* = −0.17; *r* = −0.25^12B^	*r* = −0.015, *r* = 0.041, *r* = −0.104^5B^			*r* = 0.29^13C^	^14A^	*r* = −0.05, *r* = −0.10^15A^
NES	Cortisol	—	—	—	—	—	—	—	—	—	—	***ρ*** **= 0.48**^16B^							
	CgA	—	—	—	—	—	—	—	—	—	—	—							
	Alpha-amylase	—	—	—	—	—	—	—	—	—	—	—	—						
EEG	Alpha power	—	—	—	—	—	—	—	—	—	—	—	—	—		***r* = −0.42**, *r* = −0.09^7C^			

Significant results in bold (*p* < 0.05). Correlations between variables that are not shown, were not tested in any of the studies.

Types of associations: (A) relationship between collapsed measures across conditions, (B) relationship separately for each condition, and (C) relationship between changes in measures between conditions. (1) Plain et al. (2021). Order: alone and difficult, alone and easy, observed and difficult, observed and easy, overall average. (2) Francis et al. (2016). (3) Francis et al. (2021). (4) Mackersie et al. (2015). HRV at baseline, SCL reactivity: *z*-scores compared with baseline. (5) Alhanbali et al. (2019). Alpha power correlations order: baseline, retention, and speech period. (6) Fiedler et al. (2021). (7) Lim et al. (2021). Order: 500-msec, 0-msec interval conditions. (8) McMahon et al. (2016). Order: 6-channel, 16-channel vocoding. (9) Miles et al. (2017). Order: 6-channel vocoding, 50% intelligibility, 16-channel and 50%, 6-channel and 80%, and 16-channel and 80%, mean correlation. (10) Seifi Ala et al. (2020). (11) Silcox and Payne (2021). Simple slopes estimates order: HighExp, HighUnexp, and LowUnexp conditions. Simple slope contrasts order: HighExp vs. HighUnexp, HighExp vs. LowUnexp, and HighUnexp vs. LowUnexp. (12) Zekveld et al. (2019). Order: baseline, during testing and after testing for the 50% and 71% conditions. (13) Muller et al. (2019). (14) Zekveld et al. (2014). Positive associations in frontal and temporal brain areas. (15) Zhou et al. (2022). Order: left LFC and left AC. (16) Kramer et al. (2016). Note that McMahon et al. (2016) and Miles et al. (2017) assessed the relationship on intraindividual level (*r* represents the mean coefficient), all other studies on inter-individual level.

AC, auditory cortex; Ampl, amplitude; BOLD, blood oxygen level dependent; BVPA, blood volume pulse amplitude; CgA, chromogranin A; EEG, electroencephalography; ERP, event-related potential; fMRI, functional magnetic resonance imaging; fNIRS, functional near-infrared spectroscopy; Freq, frequency; HF, high frequency; HR, heart rate; HRV, heart rate variability; ICG, impedance cardiogram; LFC, lateral frontal cortex; M, mean; MPD, mean pupil dilation; NES, neuroendocrine system; N.S., none significant; PEP, pre-ejection period; PPD, peak pupil dilation; RMSSD, root mean square of successive differences; SCL, skin conductance level; SCR, skin conductance response; SSE, simple slope estimate; SSCDE, simple slope contrast difference estimate.

If correlations were found between physiological outcome measures, these were moderate (Schober et al. 2018) at best (Table 4). The strongest correlation coefficients were a negative correlation between PEP and HF-HRV (*r* = −0.43) (Plain et al. 2021) and a positive correlation between CgA and cortisol (*ρ* = 0.48) in a specific condition (Kramer et al. 2016). It is important to note that these measures were from the same modality: the cardiovascular and neuroendocrine system, respectively. The highest correlation between measures from different modalities was between SCL and baseline HF-HRV (*r* = −0.41) (Mackersie et al. 2015) followed by alpha-amylase and PPD (*r* = −0.26) (Zekveld et al. 2019). Notably, together with the negative relationship between MPD and N400 amplitude found by Silcox and Payne (2021), these were the only significant correlations found between measures from different modalities among the included studies.

### Subjective Ratings

Fifteen of the 28 articles included subjective measurements, such as subjective effort or subjective performance. Results on subjective performance and subjective difficulty were also included, because these can bias the subjective reporting on listening effort (Moore & Picou 2018). The most frequently used scale was the NASA Task Load Index (TLX) (Hart & Staveland 1988), a six-item instrument covering mental demand, physical demand, temporal demand, perceived performance, effort, and frustration. This tool was used in seven studies, usually applying the abbreviated version. Other subjective measurements included the Visual Analog Scale of Fatigue (VAS-F) (Lee et al. 1991) and Rating Scale for Mental Effort (Zijlstra 1993), both applied once. Other studies (seven) either used a categorical scale (for example, effort scale categorical units [Krueger et al. 2017]) or a standard numerical scale from 0 to 10 or 100 asking participants to rate effort, difficulty, and/or performance. Two studies also included questions regarding the participant’s tendency to give up. Lastly, 10 of the 15 studies also included correlation analyses between the subjective and physiological measures. Of these, only four studies reported significant correlations, which were weak to moderate. Alhanbali et al. (2019) found negative correlations between the VAS-F (on fatigue and energy) and MPD (*r* = −0.25), VAS-F and PPD (*r* = −0.23), and VAS-F and alpha power during the retention phase (*r* = −0.20) of the digits in noise task. These physiological measures did not correlate with the NASA-TLX subscales. Kramer et al. (2016) found, among others, a positive correlation between PPD and subjective performance (*ρ* = 0.60) and a negative correlation between PPD and the tendency to give up (*ρ* = −0.67) for the 50% intelligibility condition. The remaining two studies found a positive correlation between perceived effort (from the NASA-TLX) and SCL (*r* = 0.67) (Mackersie & Cones 2011), a positive correlation between self-reported difficulty and PPD (*r* = 0.60), and negative correlations between self-reported difficulty and cerebral oxygenation in the left auditory cortex (*r* = −0.23) and lateral frontal cortex (*r* = −0.32) (Zhou et al. 2022). The other six studies did not report any significant correlations between the physiological and subjective measures (Mackersie et al. 2015; Seeman & Sims 2015; Francis et al. 2016, 2021; Muller et al. 2019; Slade et al. 2021).

### Quality Assessment

Results from the quality assessment using the Appraisal tool for Cross-Sectional Studies (Downes et al. 2016) can be found in Table 5. Questions regarding non-responders were all answered negatively or unknown, as there were either no non-responders or they were not discussed. Sample size was justified with a power analyses or based on previous studies in 11 studies and a sample size justification was not applicable in two studies, as these were pilot studies (Kramer et al. 2016; Mackersie & Kearney 2017). Target population was clearly defined in most studies (25), but only half of the studies (14) reported recruitment details, including the populations where participants were recruited from. The latter was again not applicable to the two pilot studies. Six studies failed to provide sufficient information regarding the methods and statistical analyses to enable replication of the study and 13 studies did not report details regarding the threshold used to determine statistical significance. Three studies failed to provide sufficiently detailed descriptive results. Five studies did not include detailed description of data analyses or did not report on all analyses that were described in the methods section. Most studies (24) described the limitations of the study, and the conclusions were not considered justified by the results in one study. Five studies did not include a statement regarding potential (funding) conflicts and one reported a potential conflict of interest. Lastly, five studies did not report obtaining ethical approval and/or participant’s informed consent.

**TABLE 5.** Quality assessment

	Alhanbali et al. (2019)	Corcoran et al. (2022)	Cvijanović et al. (2017)	Fiedler et al. (2021)	Francis et al. (2021)	Francis et al. (2016)	Giuliani et al. (2020)	Haro et al. (2022)	Hjortkjaer et al. (2020)	Kramer et al. (2016)	Lim et al. (2021)	Mackersie and Calderon-Moultrie (2016)	Mackersie and Cones (2011)	Mackersie and Kearney (2017)	Mackersie et al. (2015)	McMahon et al. (2016)	Miles et al. (2017)	Muller et al. (2019)	Plain et al. (2021)	Richter (2016)	Seeman and Sims (2015)	Sendesen et al. (2023)	Seifi Ala et al. (2020)	Silcox and Payne (2021)	Slade et al. (2021)	Zekveld et al. (2014)	Zekveld et al. (2019)	Zhou et al. (2022)
1. Were the aims/objectives of the study clear?	Y	Y	Y	Y	Y	Y	Y	Y	Y	Y	Y	Y	Y	Y	Y	Y	Y	Y	Y	Y	Y	Y	Y	Y	Y	Y	Y	Y
2. Was the study design appropriate for the stated aim(s)?	Y	Y	Y	Y	Y	Y	Y	Y	Y	Y	Y	Y	Y	Y	Y	Y	Y	Y	Y	Y	Y	Y	Y	Y	Y	Y	Y	Y
3. Was the sample size justified?	Y	**N**	**N**	**N**	**N**	**N**	Y	**N**	**N**	NA	**N**	**N**	Y	NA	Y	**N**	**N**	**N**	Y	**N**	Y	Y	**N**	Y	Y	**N**	Y	Y
4. Was the target/reference population clearly defined?	Y	Y	Y	Y	Y	Y	Y	**N**	Y	Y	Y	Y	Y	**N**	Y	Y	Y	Y	Y	Y	**N**	Y	Y	Y	Y	Y	Y	Y
5. Was the sample frame taken from an appropriate population base so that it closely represented the target/reference population under investigation?	Y	U	Y	Y	U	Y	U	U	U	NA	Y	U	U	NA	U	U	U	U	Y	Y	Y	Y	Y	Y	U	Y	Y	Y
6. Was the selection process likely to select participants that were representative of the target population under investigation?	Y	U	Y	Y	U	Y	Y	U	U	NA	Y	U	U	NA	U	U	U	U	Y	Y	Y	Y	Y	Y	U	Y	Y	Y
7. Were measures undertaken to address and categorize non-responders?	N	N	N	N	N	N	N	N	N	N	N	N	N	N	N	N	N	N	N	N	N	N	N	N	N	N	N	N
8. Were the risk factor and outcome variables measured appropriate to the aims of the study?	Y	Y	Y	Y	Y	Y	Y	Y	Y	Y	Y	Y	Y	Y	Y	Y	Y	Y	Y	Y	Y	Y	Y	Y	Y	Y	Y	Y
9. Were the risk factor and outcome variables measured correctly using measurements that had been trialed, piloted or published previously?	Y	Y	Y	Y	Y	Y	Y	Y	Y	Y	Y	Y	Y	Y	Y	Y	Y	Y	Y	Y	Y	Y	Y	Y	Y	Y	Y	Y
10. Is it clear what was used to determined statistical significance and/or precision estimates?	Y	**N**	Y	**N**	**N**	Y	Y	**N**	Y	Y	**N**	**N**	**N**	**N**	Y	Y	Y	Y	**N**	**N**	Y	Y	Y	Y	**N**	**N**	**N**	Y
11. Were the methods sufficiently described to enable them to be repeated?	Y	Y	Y	Y	Y	Y	Y	**N**	Y	Y	Y	**N**	Y	**N**	Y	Y	Y	**N**	Y	Y	Y	**N**	Y	Y	**N**	Y	Y	Y
12. Were the basic data adequately described?	Y	Y	Y	Y	Y	Y	Y	Y	Y	Y	Y	Y	Y	**N**	**N**	Y	Y	Y	Y	Y	Y	**N**	Y	Y	Y	Y	Y	Y
13. Does the response rate raise concerns about non-response bias?	U	U	U	U	U	U	U	U	U	U	U	U	U	U	U	U	U	U	U	U	U	U	U	U	U	U	U	U
14. If appropriate, was information about non-responders described?	NA	NA	NA	NA	NA	NA	NA	NA	NA	NA	NA	NA	NA	NA	NA	NA	NA	NA	NA	NA	NA	NA	NA	NA	NA	NA	NA	NA
15. Were the results internally consistent?	Y	Y	Y	Y	Y	Y	Y	Y	Y	Y	Y	Y	Y	Y	Y	Y	Y	Y	Y	Y	Y	Y	Y	Y	Y	Y	Y	Y
16. Were the results presented for all the analyses described in the methods?	Y	Y	Y	Y	Y	Y	Y	Y	Y	Y	Y	U	U	U	**N**	Y	Y	Y	Y	Y	Y	Y	Y	Y	Y	Y	U	Y
17. Were the authors’ discussions and conclusions justified by the results?	Y	Y	Y	Y	Y	Y	Y	Y	Y	Y	Y	Y	Y	Y	Y	Y	Y	Y	Y	Y	Y	**N**	Y	Y	Y	Y	Y	Y
18. Were the limitations of the study discussed?	Y	Y	Y	Y	Y	**N**	Y	Y	Y	Y	Y	Y	Y	**N**	Y	Y	Y	**N**	Y	**N**	Y	Y	Y	Y	Y	Y	Y	Y
19. Were there any funding sources or conflicts of interest that may affect the authors’ interpretation of the results?	N	N	U	**Y**	U	N	N	N	N	N	N	N	U	N	N	N	N	N	N	N	N	N	N	U	N	U	N	N
20. Was ethical approval or consent of participants attained?	Y	Y	Y	Y	Y	Y	Y	Y	Y	Y	Y	**N**	**N**	**N**	Y	Y	**N**	Y	Y	Y	Y	**N**	Y	Y	Y	Y	Y	Y

N, no; NA, not applicable; U, unknown; Y, yes.

## DISCUSSION

In this systematic review, we aimed to identify studies on listening effort that simultaneously measured multiple physiological responses to task demand, as this allowed for comparisons between these measures within the same study. The two main research questions were: (1) what is the effect of changes in auditory task demand on simultaneously acquired physiological measures? and (2) what is the relationship between the responses in these physiological measures? Later, we will discuss results pertaining to each question in more detail as well as other considerations and future directions.

### Effect of Auditory Task Demand Manipulation on Physiological Measures

Overall, most of the physiological measures either show a consistent effect of auditory task demand manipulations in the expected direction or show no effect at all (Table 3). MPD, PPD, and SCL are quite consistently affected by auditory task demand, as compared with the EEG and endocrine measures that were collected in the same studies. In addition, the studies that applied SNS or PNS-specific measures consistently indicate an increase of SNS activity and a decrease of PNS activity with increasing auditory task demand. For example, if an effect was found, PEP tended to decrease (Richter 2016; Slade et al. 2021) and EDA measures generally tended to increase (Mackersie & Cones 2011; Seeman & Sims 2015; Francis et al. 2016, 2021; Mackersie & Calderon-Moultrie 2016; Cvijanović et al. 2017; Mackersie & Kearney 2017; Giuliani et al. 2020) with increasing auditory task demand, both indicating an increase in SNS activity. Conversely, in other studies, HRV tended to decrease with increasing auditory task demand (Mackersie et al. 2015; Seeman & Sims 2015; Mackersie & Calderon-Moultrie 2016; Mackersie & Kearney 2017), indicating a decrease in PNS activity. The CNS measures indicated a generally similar pattern of increased effort as a result of an increase in auditory task demand. However, Table 3 indicates that there is an apparent inconsistency in the effect of auditory task demand changes on EEG alpha power, especially in contrast to the simultaneously measured pupil dilation. Alpha power changes are thought to reflect active inhibition of irrelevant information (such as interfering noise), which would result in an increase in alpha power with increasing masker levels (Strauß et al. 2014). On the other hand, alpha power has also been found to decrease with task demand (Klimesch 1997). Specifically, alpha power decreases in brain regions that are activated during the task, whereas alpha power increases in brain regions associated with irrelevant processes (Klimesch 2012). These discrepancies in alpha power reactivity could be due to differences in factors such as selection of EEG channels or stimulus type. Other research investigating alpha power and listening effort that were not included in the current review also show inconsistent effects of auditory task demand (Obleser et al. 2012; Petersen et al. 2015; Ala et al. 2022). Lastly, although endocrine factors and fEMG were only applied in a few of the included studies, these methods do not appear to be sufficiently sensitive to capture effects of auditory task demand manipulations. Endocrine factors are dependent on many factors, such as the circadian rhythm, timing of the peak response after stimulus onset, and quality of the measurements (Hillebrand et al. 2021). In addition, these factors provide a single measurement per time point, as opposed to the continuous recordings during stimulus presentations for the majority of the other physiological measures. Facial EMG activity depends on which muscle is recorded, is relatively difficult to measure as the electrodes need very precise placement (Van Boxtel & Jessurun 1993), and may also capture facial expressions unrelated to effort (Künecke et al. 2014). Only Mackersie and Cones (2011) found an increase in frontalis fEMG activity from medium to high task demand. However, they also stated that an effect of task demand was not found within subjects, due to high variability.

### Relationship Between Physiological Measures

The reviewed studies indicated mostly nonsignificant and weak relationships between the physiological measures, and moderate at best. Weak relationships between measurements related to listening effort have been reported as well by Shields et al. (2023). They identified eight studies that included correlation analyses between distinct physiological measures, which are included in the current review as well. They concluded that only 16.3% of the correlation analyses resulted in a significant relationship between the physiological measures (Shields et al. 2023). In the current review, the strongest reported association was between measures within the same modality (SCR frequency and amplitude) and the strongest (significant) association across modalities was between SCL and PPD. However, the notion that physiological measures (from different modalities) relate to listening effort and should therefore correlate would suggest that changes in listening effort uniformly affect different organs of the body. The weak and lack of correlations could indicate that this is not the case and that effort changes are reflected differently between various modalities. However, it is also important to consider possible alternative reasons for the weak relationships, such as differences in sensitivity and latencies of the measures to changes in task demand.

The most frequently assessed relationship is that between EEG alpha power and pupillometry measures (in seven of the included studies), but none of these relationships were significant. This might also be explained by their physiological and methodological differences. Pupil dilation has been thought to reflect the effort evoked by listening to the target stimulus, while alpha power has been hypothesized to reflect inhibition of irrelevant stimuli or noise. In addition, alpha power and pupil dilation effects may be on different timescales or have different effect latencies. Seifi Ala et al. (2020) attempted to take the timescales into account, but still found no significant relationship. As discussed previously, alpha power is relatively sensitive to changes in auditory task demand (Table 3), but does not show the same consistency as pupil dilation results, supporting a different influence of changes in task demand and/or the resulting effort.

### Relationship Between Physiological and Subjective Measurements

Similar to the relationships between physiological measures, the relationships between subjective and physiological measures were generally weak or absent, consistent with previous studies (Zekveld et al. 2010; Wöstmann et al. 2015; Holube et al. 2016; Lau et al. 2019). It is difficult to subjectively rate listening effort, especially at very high difficulty levels. At these levels, the task becomes impossible, and the tendency to give up increases as well. This is often reflected by physiological reactivity which decreases at these levels (Zekveld & Kramer 2014; Decruy et al. 2020). In contrast, subjective effort ratings are often still high at these levels. In addition, when participants report their subjective effort, they tend to be influenced by their subjective levels of performance and difficulty (McGarrigle et al. 2014; Moore & Picou 2018; Francis & Love 2020). Moore and Picou (2018) found that mental effort ratings are biased by performance when performance differs between conditions, but when performance is comparable between conditions, the ratings coincided with the expected mental effort. How a person rates their subjective effort can also be influenced by factors such as hearing acuity (van Esch et al. 2013). Considering the lack of strong associations between subjective and physiological measures of listening effort, the physiological measures may not represent how a person subjectively experienced listening effort during the listening task, depending on the (wording of the) questions and experimental design. These issues should be taken into account when interpreting subjective ratings collected in a listening task.

### Knowledge Gaps and Recommendations for Future Studies

The majority of the included studies used tasks and/or conditions that are on the high end of the performance scale (>70% performance, Table 2). In addition, the studies that used adaptive tests included conditions with a narrow performance range, for example around 50% correct. The reason for the unequal distribution of previously applied conditions might be that higher intelligibility levels better reflect daily life conditions (Smeds et al. 2015). Also, at high intelligibility levels, performance might be at ceiling, which makes the addition of another measure valuable as this provides a means to demonstrate the effect of factors still affecting listening effort. Tables 2 and 3 together indicate that conditions with relatively high performances were predominantly used in studies that showed no effect of auditory task demand on the physiological measures of interest. When comparing task conditions with high performance and relatively low effort levels, the physiological measure may not be sensitive enough to show a significant effect. To get a better understanding of the (differential) sensitivity of physiological responses to changes across the full range of auditory task demand, it is important to also include very difficult and/or even (almost) impossible tasks, as well as moderate difficulties. Moreover, to further support the FUEL, it is necessary to explore the physiological responses across a wider range of the psychometric curve. Considering the non-monotonic relationship between task demand and effort, the physiological reactivity may be similar when comparing conditions with low and high-performance levels, so it is recommended to include a variety of performance levels. Furthermore, physiological measures may differ in their sensitivity to changes in either the low, mid, or high-performance range, or the shape of the non-monotonic relationship may differ between measures. For example, Decruy et al. (2020) showed that alpha power peaked between 60% and 80% intelligibility. Using fNIRS, Lawrence et al. (2018) found the activation in the left inferior frontal cortex to peak around 25 to 50% intelligibility and decrease again as the intelligibility further increased. Lastly, pupil dilation has been shown to peak around 50% intelligibility (Ohlenforst et al. 2017; Wendt et al. 2018). In addition, it is also possible that for some of the studies, absent effects might be influenced by small sample sizes and low power. As shown from the quality assessment (Table 5), more than half of the included studies did not justify their sample size based on a priori power analyses and their sample sizes were relatively small. Therefore, more studies are necessary to explore the full relationship of auditory task demand and the physiological measures using sufficiently large sample sizes.

Physiological measures of listening effort have been applied simultaneously in a considerable number of studies, but several combinations have not been used yet. For example, EEG alpha power has not been combined with HRV, PEP or cortisol. In addition, pupil dilation has been used most frequently as a measure of listening effort, but has rarely been applied in combination with an SNS or PNS measure. Although Alhanbali et al. (2019) and Giuliani et al. (2020) measured pupil response in combination with EDA, the analyses performed in those studies do not allow conclusions about the relationship between the two measures in response to changes in auditory task demand. The first study did not include SCL in the correlation analyses due to poor reliability and the latter did not test for any associations. Therefore, it also remains unclear whether changes in pupil dilation with auditory task demand are associated with (increased) SNS activation, PNS withdrawal, or a combination of both and to what extent.

The study results could not be directly compared with each other in a meta-analysis. Among the included studies, there was a great variety of types of physiological measures applied. Even if the same measures were used, the studies often differed in measurement methods and tools, signal cutoffs, pre-processing strategies, type of baseline, or different parameters were analyzed. Furthermore, the studies widely differed in the manipulations of auditory task demand and stimulus types. One of the advantages of measures like PEP, HRV, and SCL is that they consistently show the same direction of effect with increasing effort and they provide a specific indication of either SNS or PNS activity. However, these measures do not seem sensitive enough to capture differences in listening effort when differences in auditory task demand are small. Pupil dilation is also consistent and more sensitive, but does not yield information on the specific contribution of the PNS and SNS. Therefore, based on the current literature, it is not yet possible to identify a physiological index or a particular combination that best captures changes in listening effort.

The quality assessment (Table 5) indicated that the overall quality of the included studies was reasonable. A significant number of the included studies lacked details regarding justification of the sample size, selection procedure of participants, or did not report a sufficient description of the participants, all adversely affecting the ability to interpret the potential generalizability of the results. To increase the ability to detect and interpret the results, it is recommended to include adequate sample sizes based on power analyses and/or previous studies and to provide sufficient details regarding the participants and inclusion procedure. In addition, many studies included young age groups, such as university students (see Supplementary Table 1 in Supplemental Digital Content 2, http://links.lww.com/EANDH/B382). Considering age is one of the strongest predictors of hearing loss (Hoffman et al. 2017) and also influences the physiological measures, it is important to also represent older participants in future studies. Moreover, in a considerable number of the studies, details of the methods or statistical analyses were missing, potentially resulting in difficulties with replicating these studies. For example, several studies did not define the sound level of the auditory stimuli. In addition, in most cases, a critical alpha level of 0.05 was assumed, but not stated explicitly. In summary, for replicability and interpretability of the results, it is important to include the relevant details on the methods and analyses.

In the current review, we showed that the physiological measures vary in their sensitivity to auditory task demand manipulations and that relationships between the measures are generally weak. Furthermore, the physiological measures display different “peaks” with auditory task demand. These results should be taken into account when choosing which measures to apply and combine, also depending on which auditory task manipulations are used to induce listening effort. Our results support Alhanbali et al. (2019) who concluded that measures of listening effort are multidimensional and should not be used interchangeably. In addition, it supports FUEL, which states that multiple mechanisms underpin listening effort, such as working memory, attention, and speed of processing in the cognitive domain (Pichora-Fuller et al. 2016). Pichora-Fuller et al. (2016) also stated that it is necessary to investigate the relationship between measures of listening effort and “whether or not it would be advantageous to combine tests.” The current review shows that the correlations between measures are weak and that it is advantageous to combine measures, depending on the research questions and study design. Also, the current review indicates that there is not yet a gold standard procedure of measuring effort, and developing such a standard might not even be feasible for all cases as the current measures might be differentially sensitive to different processes or manipulations of interest. Therefore, we want to highlight the need for standardized methods for each measure and clear justification of the chosen physiological measures. Last, an additional important consideration regarding the physiological responses and their relationships is the application of intra-individual or inter-individual analyses, as this can considerably affect results (LoTemplio et al. 2021; Rab & Admon 2021). For an overview of recommendations regarding the use of multiple physiological measures, see also Richter et al. (2023).

## LIMITATIONS

A limitation of the current review is that the summary of the effects of increasing auditory task demand on the physiological measures (Table 3) does not take into account that the non-monotonic relationship may differ between measures. As the details of these functions for each measure are currently partly unknown, it was not yet possible to illustrate the physiological responses over the full range of the hypothesized non-monotonic relationships. It is therefore advised to take into account the performance levels in each study (Table 2) in addition to the summary provided by Table 3. One of the caveats posed by some of the included studies is that the physiological measures were not tested simultaneously. For example, Zhou et al. (2022) were unable to use pupillometry and fNIRS simultaneously due to limitations of using fNIRS (participants were not allowed to speak during the task). Similarly, saliva samples (for example to measure CgA or cortisol) are usually retrieved once before, during, and after a task, which may affect its comparability with a continuously assessed measure, such as pupil dilation (Kramer et al. 2016; Zekveld et al. 2019). Second, only a few of the studies tested participants who were hard-of-hearing. Therefore, we did not summarize these studies separately, although hearing loss can influence the physiological responses to auditory task demand manipulations. For example, Kramer et al. (2016) found a smaller PPD increase in the hard-of-hearing group compared with normal hearing participants for decreasing SNR, but no effect of group on CgA or cortisol was found. Mackersie et al. (2015) found a decreased HRV in participants with hearing impairment but not in participants with normal hearing for the difficult conditions, as well as a higher SCL reactivity for hard-of-hearing participants (see Supplementary Table 1 in Supplemental Digital Content 2, http://links.lww.com/EANDH/B382, which provides a detailed overview of included the articles). Last, and as described earlier, the included studies varied greatly in procedures to manipulate auditory task demand, several measures have not yet been combined in the same studies, and the lack of consistent relations between measures limit the ability to make clear comparisons.

## CONCLUSIONS

The current review assessed the effect of auditory task demand manipulations on simultaneously measured physiological responses and the relationship between these measures. Auditory task demand was mostly manipulated by changing the auditory quality of the stimuli. Most of the studies included conditions with relatively high-performance levels and normally hearing participants. Except for alpha power, all physiological measures either showed a consistent effect in the expected direction or no effect to auditory task demand manipulations. Within the included studies, most measures were not equally sensitive to task demand. In addition, the associations between the physiological measures were mostly non-significant or weak. These findings support the argument that these measures differ in their sensitivity to changes in listening effort and/or tap into different aspects of listening effort. Therefore, this review supports the FUEL and highlights the need for taking into account a multimodal approach when applying physiological measures in research to listening effort. Moreover, we highlighted a number of considerations for future studies, such as the need for better standardized methods and the use of a wider range of intelligibility levels.

## ACKNOWLEDGMENTS

This work was supported by funding from ZonMw (grant number 09120011910029).

## Supplementary Material
