# Supplementary material for: A Multimodal Approach to Measuring Listening Effort: A Systematic Review on the Effects of Auditory Task Demand on Physiological Measures and Their Relationship
Source: Ear Hear. 2024 Jun 17;45(5):1089–106. doi: 10.1097/AUD.0000000000001508 (PMC11325958; doi:10.1097/AUD.0000000000001508)
Supplement: Supplementary file 1 [file aud-45-1089-s001.pdf]

Supplementary Material to:

Manuscript title: A multimodal approach to measuring listening effort: a systematic review on the effects of auditory load on physiological measures and their relationship.

Appendix

General search terms used in Pubmed, PsycInfo, and Web of Science. Iterations of all different combinations were performed. Search terms that were not applied as MeSH terms, were specified to be sought in the abstract and title.

(("Heart Rate"[MeSH Terms]) OR ("Blood Pressure"[MeSH Terms]) OR ("Electrocardiography"[MeSH Terms]) OR ("heart rate") OR ("pulse rate") OR ("blood pressure") OR ("heart period"))

AND (

(("Galvanic Skin Response/physiology"[MeSH Terms]) OR ("skin conductance") OR (electrodermal)) OR

(("heart rate variability") OR (HRV) OR (“respiratory sinus arrythmia”) OR (RSA) OR (SDNN) OR (RMSSD) OR ("Respiratory Sinus Arrhythmia"[MeSH Terms])) OR

(("pre-ejection period") OR (PEP)) OR

((hormone*) OR (endocrine) OR ("Chromogranin A/metabolism"[MeSH Terms]) OR (“Hydrocortisone/metabolism"[MeSH Terms]) OR (“alpha-Amylases/metabolism*"[MeSH Terms])) OR

((pupil*) OR (pupillomet*)) OR

(("Electroencephalography"[MeSH Terms]) OR (Electroencephalogr*) OR (“alpha power”) OR (“beta power”) OR (“delta power”) OR (“theta power”) OR (“N400 amplitude”)) OR

((MEG) OR (magnetoencephalogr*)) OR

((EMG) OR (fEMG) OR (Electromyography[MeSH Terms]) OR (“facial electromyogra*”)) OR

((fNIRS) OR (“functional near-infrared spectroscopy”)) OR

((fMRI) OR (“functional magnetic resonance imaging”) OR (“BOLD response”) OR ("Magnetic Resonance Imaging/methods*"[MeSH Terms]))

) AND

(("listening effort") OR (“Listening effort”[MeSH Terms]) OR (“Speech Intelligibility” [MeSH Terms]) OR (“effortful listening”) OR ("listening fatigue") OR ("listening comprehension") OR ("listening demand") OR ("sentence recognition") OR ("speech recognition") OR ("speech perception") OR ("auditory load") OR (intelligibility))
