# Supplementary material for: A Multimodal Approach to Measuring Listening Effort: A Systematic Review on the Effects of Auditory Task Demand on Physiological Measures and Their Relationship
Source: Ear Hear. 2024 Jun 17;45(5):1089–106. doi: 10.1097/AUD.0000000000001508 (PMC11325958; doi:10.1097/AUD.0000000000001508)
Supplement: Supplementary file 2 [file aud-45-1089-s002.pdf]

Supplementary Material to:

Manuscript title: A multimodal approach to measuring listening effort: a systematic review on the effects of auditory task demand on physiological measures and their relationship.

Supplementary Table 1. Summary of included studies, listing details about the subjects and a short description of the relevant measures, tasks and findings. The footnotes in the column with the relevant findings (effects) refer to the test results listed in the next column.

	Reference	Subjects (age in years)	Relevant measures	Task(s)	Relevant findings: Effects	Test results (effects)	Relevant findings: Associations
1	Alhanbali et al. (2019)	N=116 (55-85yrs (M=70); 50.3% males).	Pupil dilation; SCL; EEG alpha activity.	Digits in noise (individually adapted SNR to reach 71% intelligibility; unmodulated background noise). 30 participants performed a retest. Pupil baseline: 1s before digit onset EEG baseline: 5s of noise before digit onset. Task: y/n question whether number on screen was presented in sequence of 6 digits.	Intelligibility exceeded 71%, and this score differed between groups[1a]. The average scores were: NH: 93% (±4), mild HI: 89% (±7), moderate HI: 87% (±6), severe HI: 82% (±6). Mean SNR for NH was -9 dB (±2), for mild HI -3 dB (±3), for moderate HI -1 dB (±5), and for severe HI +4 dB (±4). **Pupil size increased** relative to baseline[1b,c]. **Alpha power increased** during digit presentation compared to baseline[1d]. Due to poor reliability of SCL[1e], SCL was not included in the FA. **FA indicated four dimensions** explaining 74% of variance: 1) performance accuracy, hearing level, SNR, and baseline alpha power; 2) MPD and PPD; 3) alpha power during digit presentation and retention; 4) self-reported effort and baseline alpha power.	**1a.** ANOVA: $F_{(3,93)}$=10.1*. Pairwise comparisons: p<0.013. **1b.** Median mean pupil size: 0.02 mm, IQR=0.08. **1c.** Median peak pupil size: 0.11 mm, IQR=0.12. **1d.** 0.5 to 4 sec: z=−2.30*. **1e.** r_s=0.48*, ICC=0.50.	SCL and alpha power during speech presentation showed a negative correlation (r=-0.252, p=0.008); SCL and PPD showed a positive correlation (r=0.209, p=0.025). All other correlations were non-significant.
2	Corcoran et al. (2022)	N=19 NH (19-33yrs (M=25.8); 57.9% males).	Pupil dilation; EEG oscillatory power.	Noise-vocoded and sine-wave synthesized sentences (NVS and SWS resp.), with correct, incorrect or without prior text presentation of the visual sentence. Task: answer whether the sentence matched the text (yes/no).	Mean performance was 95.8% (±1.3) for SWS and 95.0% (±1.4) for NVS sentences. **Delta[2a] and theta[2b] power were higher** for NVS than SWS sentences. **Alpha[2c] and beta[2d] power did not differ** between the stimuli. After presenting sentences textually (either congruent or incongruent), delta[2e] and alpha[2f] power increased during listening compared to listening without prior text. Similarly, based on cluster based permutation analyses, **pupil dilation increased** after both congruent[2g] and incongruent[2h] prior information compared to no information. After correct texts only, theta activity decreased during listening[2i].	**2a.** $\chi^2_{(1)}$=5.87, p=0.015. **2b.** $\chi^2_{(1)}$=5.53, p=0.019. **2c.** $\chi^2_{(1)}$=1.78, p=0.182. **2d.** $\chi^2_{(1)}$=1.68, p=0.194. **2e.** Congruent vs no prior information: z-ratio=2.37, p=0.047; Incongruent vs no prior information: z-ratio=3.91***. **2f.** Congruent vs no prior information: z-ratio=3.60***; Incongruent vs no prior information: z-ratio=5.47***. **2g.** SWS:[0.7, 6.4]s; NVS: [0.6, 11]s. **2h.** SWS: [3.8, 11]s; NVS: [0.6, 11]s. **2i.** Congruent vs no prior information: z-ratio=2.64, p=0.022; Congruent vs incongruent prior information: z-ratio=4.27***.	

3	Cvijanović et al. (2017)	N=40 NH (18-30yrs; sex not reported).	SCL; SCR (frequency); HRV (LF, LF/HF ratio).	Speech in noise in communication setting (silent, +6dB, -6 dB SNR; non-stationary cafeteria noise); while solving puzzles in pairs to stimulate communication.	**SCL increased with decreasing SNR**[3a], for silent vs. -6 dB SNR[3b]. No differences between the silent and +6 dB SNR[3c] and the +6 dB SNR and -6 dB SNR[3d] conditions. **SCR frequency did not differ between conditions**[3e]. HRV (LF[3f] and LF/HF ratio[3g]) did not differ between conditions.	**3a.** $\chi^2_{(2)}$=7.28*. **3b.** *p*<0.05. **3c.** *p*=0.61. **3d.** *p*=0.46. **3e.** $\chi^2_{(2)}$=2.95, *p*=0.23. **3f.** $\chi^2_{(2)}$=1.08, *p*=0.58. **3g.** $\chi^2_{(2)}$=1.85, *p*=0.4.	
4	Fiedler et al. (2021)	N=22 HI (40-80yrs (M=67±11.2); sex not reported).	Pupil dilation; EEG parietal alpha power.	Speech (news clips; female vs. male talker) in noise (hearing aid noise reduction [NR] on/off; +8, +3 dB SNR; four talker babble). Task: two-choice comprehension question after each clip. Baseline interval: 5 seconds of noise before the start of each trial.	NR[4a] and SNRs[4b] had no effect on performance, but there was an (unintentional) effect of talker[4c]: performance was better for the female than the male talker. Overall, pupil size decreased[4d] and parietal alpha power increased[4e] during listening, compared to baseline. NR decreased average pupil size[4f], but there was no effect on parietal alpha power[4g]. The effect of NR on pupil size was stronger for +3 dB SNR compared to +8 dB SNR (interaction)[4h]. **No effect of SNR on pupil size**[4i] or **parietal alpha power**[4j] was found. Pupil size was lower for the female compared to male talker[4k]. There was also an **interaction between SNR and talker for pupil size**[4l]. **Parietal alpha power was higher for the female compared to male talker**[4m].	**4a.** β=0.22, SE=0.15, $t_{(1671)}$=1.5, *p*=0.14. **4b.** β=0.27, SE=0.27, $t_{(1671)}$=3.4, *p*=0.067. **4c.** β=0.79, SE=0.15, $t_{(1671)}$=29.5***. **4d.** β=–0.16, SE=0.037, $t_{(1172)}$=–4.24***. **4e.** β=50.2, SE=9.1, $t_{(1507)}$=5.52***. **4f.** β=–0.046, SE=0.008, $t_{(1172)}$=–5.66***. **4g.** β=5.85, SE=3.92, $t_{(1507)}$=1.4, *p*=0.136. **4h.** β=0.038, SE=0.0164, $t_{(1172)}$=2.31, *p*=0.021. **4i.** β=–0.013, SE=0.008, $t_{(1172)}$=–1.6, *p*=0.107. **4j.** β=5.9, SE=3.91, $t_{(1507)}$=1.53, *p*=0.127. **4k.** β=-0.037, SE=0.008, $t_{(1172)}$=-4.56***. **4l.** β=0.033, SE=0.016, $t_{(1172)}$=2.05, *p*=0.041. **4m.** β=9.2, SE=3.90, $t_{(1507)}$=2.35, *p*=0.019.	Linear mixed-effects models showed no main effect of parietal alpha power on pupil size (β=–0.005, SE=0.004, t_{1090}=–1.23, p=0.22) or vice versa (β=–2.8, SE=2.45, t_{1089}=–1.15, p=0.25), which indicates that these measures are not correlated.
5	Francis et al. (2021)	N=35 NH (18-71yrs (M=37.14); 34.3% males; mean PTA=0.58 (±4.56)).	EMG; BVPA; SCL; SCR (frequency); HP.	Speech (words) in noise and non-native accented speech (adjusted SNR to match performance on the comprehension questions between conditions). Measurements compared to resting baseline (log ratios). Personality Dimension 1 (as derived from PCA) related to extraversion, agreeableness, and neuroticism traits. Personality Dimension 3	The individually adjusted SNR was on average -2.05 (±3.34) dB. Performance for accented speech was 77.9% (±13.4) and in noise 78.9% (±13.0). **BVPA and EMG did not differ in both conditions** compared to baseline. **SCL increased** compared to baseline[5a]. SCL did not differ between conditions, but hearing thresholds interacted with condition[5b]: **SCL decreased with higher thresholds**. **SCR frequency increased** compared to baseline[5c] and more in noise[5d] compared to accented speech. Personality Dimension 1 was related to SCR[5e]. HP decreased compared to baseline[5f], but did not differ between conditions. Personality Dimension 3	**5a.** average log ratio=0.023, SD=0.052. **5b.** $F_{(1,21.96)}$=5.33, *p*=0.031. **5c.** average log ratio=0.33, SD=0.32. **5d.** $F_{(1,5.39)}$=24.68, p=0.003. **5e.** $F_{(1,16.69)}$=5.05, p=0.038. **5f.** average log ratio= -0.006, SD=0.014. **5g.** $F_{(1,22)}$=7.01, *p*=0.015. **5h.** $F_{(1,22)}$=5.81, *p*=0.025. **5i.** $F_{(1,21.87)}$=5.54, *p*=0.028. **5j.** $F_{(1,21.80)}$=7.44, *p*=0.012.	SCL and SCR correlated (*r*=0.30***), but BVPA did not correlate with SCL (*r*=-0.052, *p*=0.378) or SCR (*r*=0.045, *p*=0.580).

				related to openness and conscientiousness traits.	contributed to an increase in HP[5g], as well as vocabulary[5h]. Interaction effects between condition and age[5i] and between condition and working memory capacity[5j].		
6	Francis et al. (2016)	N=14 NH (20-32yrs (M=26.0); 21.4% males).	SCR (frequency and amplitude);<br>Blood pulse amplitude;<br>Pulse rate.	Speech (sentences) in quiet and noise (-8 dB SNR; distortion, energetic masking, informational masking).<br>Task: sentence repetition.	Scores on sentence repetition were 99.7% (±0.7) for unmasked natural speech, 90.3% (±5.1) for speech-shaped noise masker, 89.4% (±4.8) for two-talker babble masker and 93.1% (±3.8) for unmasked synthetic speech condition.<br>Performance was higher[6a] for quiet compared to noise and lower for the two-talker babble compared to synthetic speech[6b]. No difference in performance between two-talker babble and synthetic speech.<br>**SCR frequency did not differ** between conditions[6c].<br>**SCR amplitude differed between conditions**[6d], with higher reactivity for two-talker babble than for speech-shaped noise[6e].<br>**Pulse rate did not differ** between conditions[6f], but decreased specifically at the end of the stimulus (~4%)[6g].<br>Blood pulse amplitude was different between conditions[6h] and between time points[6i]. Between conditions, no pairwise differences were significant. Overall, **blood pulse amplitude decreased during stimulus presentation.**	**6a.** $F_{(3,39)}$=44.47***, η^2_p=0.77.<br>**6b.** *p*=0.04.<br>**6c**. $F_{(3,39)}$=2.03, *p*=0.13, η^2_p=0.14.<br>**6d.** $F_{(3,36.2)}$=3.02, *p*=0.04, η^2_p=0.21.<br>**6e.** p_{adj}=0.031.<br>**6f**. $F_{(3,39)}$=1.31, *p*=0.28, η^2_p=0.09.<br>**6g.** $F_{(10,520)}$=4.65***, η^2_p=0.08.<br>**6h.** $F_{(3,39)}$=3.52, *p*=0.02, η^2_p=0.21.<br>**6i.** $F_{(10,520)}$=59.06***, η^2_p=0.53.	No significant (uncorrected) Pearson correlations were found. Coefficients not reported.
7	Giuliani et al. (2020)	N=20 NH (18-50yrs (M=28.3); 30% males).	Pupil dilation;<br>SCR amplitude.	Speech (sentences) in quiet and noise (0, -3, -5 dB SNR; stationary noise); measurements and tests were completed twice during two sessions.<br>Task: sentence repetition.	Performance differed between each SNR condition[7a]: quiet > 0 dB SNR > -3 dB SNR > -5 dB SNR.<br>SNR influenced the grand mean change of pupil diameter[7b]: **pupil size increased with decreasing SNR**. Reliability was fair (ICC=0.48).<br>**SNR did not affect the mean SCR amplitude** during speech presentation[7c]. SCR amplitude reliability during speech presentation was fair (ICC=0.45).<br>SNR influenced the mean SCR amplitude during the response window[7d]: **SCR amplitude increased from quiet to -3 dB SNR**[7e]. SCR amplitude reliability during the response window was fair (ICC=0.41). Word recognition performance affected SCR amplitude[7f]: **SCR amplitude was higher for participants scoring ≤50% compared to >71% correct**[7g].	**7a.** $F_{(3,460)}$=1055.717*** [corrected α=0.008].<br>All pairwise tests***.<br>**7b.** $F_{(3,135.981)}$=6.129*** [corrected α=0.008].<br>**7c.** $F_{(3,1555.860)}$=0.794, *p*=0.794.<br>**7d.** F(3,1977.509)=3.096, *p*=0.026.<br>**7e.** *p*=0.002.<br>**7f.** F(2,1977.666)=3.600, *p*=0.027.<br>**7g.** *p*=0.007.	
8	Haro et al. (2022)	N=10 NH (age not reported; 50% males).	Peak pupil dilation;<br>EEG parietal alpha power.	Auditory attention task (3 conditions: at-will attention switch [AWAS], directed attention switch [DAS], sustained attention [SA; no switch]). Two simultaneously presented audiobook clips.	Mean performance was 58% for AWAS, 51% for DAS, and 58% for SA. There was a main effect of conditions on accuracy[8a], but no pairwise differences.<br>Condition influenced PPD around the time of attention switching[8b]: **PPD was higher for AWAS than SA**[8c].	**8a.** $F_{(2,18)}$=4.247, *p*=0.0308.<br>**8b.** $F_{(2,18)}$=7.668, *p*=0.0039.<br>**8c.** *p*=0.034.<br>**8d.** $F_{(2,18)}$=5.715, *p*=0.012.<br>**8e.** *p*=0.016.<br>**8f.** *p*=0.0056.<br>**8g.** *p*=0.0056, FPR of 0.05.	

				Accuracy scores for comprehension questions.	Condition had an effect on minimum parietal alpha ERSP around the time of attention switching[8d]: minimum parietal **alpha ERSP was lower for AWAS than SA**[8e], also in the centrotemporal[8f] and parieto-occipital[8g] regions**. In the frontal regions, minimum alpha ERSP was lower for DAS compared to SA**[8h].	**8h.** p=0.0041, FPR of 0.05.	
9	Hjortkjaer et al. (2020)	N=22 NH (19-28yrs (M=24±3); 72.7% males).	Pupil dilation;<br>EEG (oscillatory power: alpha and theta power; temporal response functions [TRF]).<br>Cluster based permutation analyses on EEG data.	Auditory N-back task with digits in noise (1-back, 2-back; +10 and 0 dB SNR; stationary background noise).	Performance was lower for 2-back compared to 1-back[9a]; no effect of SNR[9b] (as intended).<br>**MPD[9c] and PPD[9d] were higher for 2-back compared to 1-back. No effect of SNR** on MPD[9e] and PPD[9f].<br>**Alpha power decreased with N-back level** in posterior and central regions, but **theta power showed no significant cluster**.<br>Alpha power decreased and theta power increased with increased N-back level at electrode Oz[9g] (alpha power) and electrode Afz[9h] (theta power)**. No effect of SNR on alpha**[9i] **and theta power**[9j].<br>For TRF, peak latency increased[9k] and peak amplitude decreased[9l] for 0 dB SNR compared to +10dB SNR. No effects of N-back conditions on peak amplitude[9m] or latency[9n].	**9a.** $F_{(1,21)}$=203.77***.<br>**9b.** $F_{(1,21)}$=0.7487, p=0.397.<br>**9c.** 0–45s: $F_{(1,11)}$=17.00, p=0.0017.<br>**9d.** $F_{(1,11)}$=20.16***.<br>**9e.** $F_{(1,11)}$=0.31, p=0.58.<br>**9f.** $F_{(1,11)}$=0.76, p=0.40.<br>**9g.** $F_{(1,18)}$=30.15***.<br>**9h.** $F_{(1,18)}$=10.88, p=0.004.<br>**9i.** $F_{(1,18)}$=1.90, p=0.18.<br>**9j.** $F_{(1,18)}$=0.29, p=0.60.<br>**9k.** $F_{(1,18)}$=20.43***.<br>**9l.** $F_{(1,18)}$=12.95, p=0.002.<br>**9m.** $F_{(1,18)}$=0.80, p=0.381.<br>**9n.** $F_{(1,18)}$=0.84, p=0.371.	
10	Kramer et al. (2016)	N=10 NH (M=53.3yrs; 30% males);<br>N=10 HI (M=50.7yrs; 20% males).	Peak pupil dilation;<br>Saliva cortisol;<br>Saliva CgA.	Speech (sentences) in quiet and noise (50% intelligibility; interfering speech).<br>Task: sentence repetition.	Mean SNR in noise was lower for NH (-12.0 (±2.5) dB) compared to HI (1.0 (±7.8) dB)[10a].<br>**PPD was higher for noise** compared to quiet[10b] for both groups. No main effect of group[10c], but group interacted with condition[10d]: **HI showed a smaller increase in PPD compared to NH**. The group effect on PPD was only in noise[10e], not in quiet[10f].<br>**Cortisol levels did not differ** between groups[10g] and conditions[10h].<br>**CgA did not differ** between groups[10i], but there was a main effect of condition[10j]: CgA levels were higher before testing compared to both the quiet condition[10k] and after testing[10l].	**10a.** p<0.001.<br>**10b.** $F_{(1,18)}$=23.5***, effect size d=0.69.<br>**10c.** $F_{(1,18)}$=2.4, p=0.14.<br>**10d.** $F_{(1,18)}$=3.6, p=0.075.<br>**10e.** $t_{(18)}$=1.9, p=0.074.<br>**10f.** $t_{(18)}$=−0.18, p=0.86.<br>**10g.** $F_{(1,17)}$=1.9, p=0.19.<br>**10h.** $F_{(3,51)}$=0.4, p=0.66.<br>**10i.** $F_{(1,14)}$=0.01, p=0.92.<br>**10j.** $F_{(3,42)}$=4.4, p=0.027.<br>**10k.** $t_{(16)}$=3.4, p=0.018.<br>**10l.** $t_{(15)}$=3.0, p=0.054 (Bonferroni corrected α=0.10).	Moderate correlation between cortisol levels and CgA levels in the quiet condition (ρ=0.48).<br>No significant correlations were found in other conditions.
11	Lim et al. (2021)	N=24 NH (18-30yrs (M=21); 33.3% males).	Pupil dilation;<br>EEG (oscillatory power, P3 ERP).	Digit recall task (single vs mixed talker; 0 ms vs 500 ms interval; sequences of 7 digits).	Recall accuracy was higher for single compared to mixed talker[11a], but no difference between intervals[11b].<br>Based on permutation based cluster tests, there was a main effect of talker on average ERPs in one cluster[11c]: the **mixed talker evoked stronger positive potential** than single talker, mostly frontocentral.<br>There was a main effect of interval: the **500 ms intervals showed stronger positive potential** than with 0 ms intervals in two clusters[11d].<br>**During digit encoding, there were no effects of talker or interval on alpha power**[11e]. **During memory**	**11a.** $\chi^2_{(1)}$=16.03***.<br>**11b.** $\chi^2_{(1)}$=0.47, p=0.50.<br>**11c.** 215–348 ms after digit presentation***.<br>**11d.** Cluster 1:0-63 ms after digit presentation, p=0.015; Cluster 2: 105-500 ms***.<br>**11e.** 0 ms intervals: all ps≥0.11; 500 ms intervals: all ps≥0.60.<br>**11f.** p=0.001.<br>**11g.** p=0.003.<br>**11h.** $F_{(1,21.8)}$=58.76***.	Pairwise correlations reported of differences between the measures in the single vs mixed talker.<br><br>*0 ms interval*<br>Δ P3d ERP and Δ alpha power (r=-.09, p=.88).<br>Δ P3d ERP and Δ pupil dilation (r=-.24, p=.27).<br>Δ pupil dilation and Δ alpha power (r=-.27, p=.21).

					**retention, alpha power was lower for mixed talker** compared to single talker[11f]. **Alpha and low beta power were higher** for 0 ms compared to 500 ms intervals[11g].<br>During digit encoding, there was a main effect of digit position[11h] on pupil dilation and an interaction between interval and digit position[11i]: the increase in **pupil dilation per digit was larger for 0 ms** than for 500 ms intervals. No main effect of talker[11j] or interval[11k] on pupil dilation, but the interaction was significant[11l].<br>**During memory retention, no effects of talker[11m] or interval[11n] were found on pupil dilation**.	**11i.** $F_{(1,20310)}$=9.88, *p*=0.0017.<br>**11j.** $F_{(1,21.8)}$=0.19, *p*=0.67.<br>**11k.** $F_{(1,21.3)}$=2.61, *p*=0.12.<br>**11l.** $F_{(1,20312)}$=5.45, *p*=0.020.<br>**11m**. $F_{(1,21.53)}$=0.57, *p*=0.46.<br>**11n.** $F_{(1,19.77)}$=0.12, *p*=0.73.	*500 ms interval*<br>Δ P3d ERP and Δ alpha power (r=-.42, *p*=.041).<br>Δ P3d ERP and Δ pupil dilation (r=.065, *p*=.77).<br>Δ pupil dilation and Δ alpha power (r=.048, *p*=.83).
12	Mackersie and Calderon-Moultrie (2016)	N=26 NH (20-35yrs (M=25.3); 34.6% males).	SCL;<br>HRV (HF).<br>Compared to resting baseline.	Speech (sentences) at normal and fast rate in noise (0 and +3 dB SNR, resp.).<br>Task: sentence repetition.	Performance did not differ between normal (80.7% correct on average) and fast rate (79.0% correct on average)[12a].<br>**SCL reactivity (i.e. increase) was higher for fast speaking** than normal speaking rate[12b].<br>**HRV reactivity (i.e. decrease) was greater for the fast rate** compared to the normal rate[12c].	**12a.** $t_{(25)}$=0.86, *p*=0.040, E_s=0.17.<br>**12b.** $t_{(25)}$=2.92, *p*=0.008, E_s=0.60.<br>**12c.** $t_{(25)}$=3.30, *p*=0.003, E_s=0.63.	
13	Mackersie and Cones (2011)	N=15 NH (24-54yrs (M=27); 13.3% males).	SCL;<br>HR;<br>EMG.	Dichotic digit task (3 conditions: low [1 digit presented in 1 ear], medium [2 different digits in each ear], hard [2 pairs of different digits in each ear]).	Performance was ~96.5-100% for all conditions.<br>**SCL[13a] and EMG[13b] activity increased** with increase of task demand.<br>**HR did not change** with task demand[13c].	**13a.** $F_{(2,28)}$=5.40**, η^2_p=0.28.<br>**13b.** $F_{(2,28)}$=4.75, *p*=0.02, η^2_p=0.25.<br>**13c.** $F_{(2,28)}$=2.13, *p*=0.14, η^2_p=0.14.	
14	Mackersie and Kearney (2017)	N=12 HI (64-88yrs (M=76); sex not reported).	SCL;<br>HRV (RMSSD).	Low vs high demand: Speech (narrative) recall without or with comprehension (in quiet and noise (+6 dB SNR); no effect of noise so data were merged).<br>Low vs high evaluative threat.	**SCL increased and HRV decreased** compared to baseline for all conditions[14a] (low vs high demand, low vs high threat).<br>Evaluative threat interacted with task demand[14b]: **SCL was higher during high demand/high threat condition**.<br>There was **no interaction effect** of evaluative threat and task demand on **HRV**[14c].	**14a.** No test results reported.<br>**14b.** $F_{(1,11)}$=7.10, *p*=0.02, η^2_p=0.39.<br>**14c.** $F_{(1,11)}$=0.78, *p*=0.39, η^2_p=0.07.	
15	Mackersie et al. (2015)	N=15 NH (24-78yrs (M=45); 33.3% males);<br>N=18 HI (22-79yrs (M=58); 50% males).	SC reactivity (z-scores compared to quiet);<br>HRV (HF).	Speech (sentences) in quiet and noise (+3, 0, -3, -6 dB SNR relative to listener's adaptive threshold; five talker babble).<br>Task: sentence repetition.	Mean adaptive thresholds differed between HI (5.2 (±0.65) dB SNR) and NH (3.5 (±0.71) dB SNR)[15a].<br>Main effect of SNR[15b] on performance, but not of hearing status[15c].<br>**SC reactivity was higher for HI** compared to NH[15d], but was **not sensitive to SNR**[15e].<br>For HRV, hearing status interacted with SNR[15f]: **HRV decreased for the two lowest SNRs in HI**, but not NH.	**15a.** $t_{(31)}$=-0=1.85, *p*=0.07.<br>**15b.** $F_{(3,93)}$=211.13***, η^2_p=0.87.<br>**15c.** $F_{(1,31)}$=1.43, *p*=0.24, η^2_p=0.04.<br>**15d.** $F_{(1,31)}$=5.20, *p*=0.03, η^2_p=0.14.<br>**15e.** $F_{(3,93)}$=1.30, *p*=0.28, η^2_p=0.04.<br>**15f.** $F_{(3,90)}$=3.62, *p*=0.016, η^2_p=0.11.	Moderate negative correlation between HRV at baseline and SC reactivity (*r*=-0.41, *p*=0.021).

16	McMahon et al. (2016)	N=16 NH (19-28yrs (M=23); 37.5% males).	Pupil dilation; EEG alpha power.	Speech (sentences) in noise (6-channel and 16-channel vocoded material with noise randomly presented between +7 to -7 dB SNR; four talker babble).<br>Task: sentence repetition.<br>LME regression model results are provided.<br>Correlations were performed on individual data.	Interaction effect between SNR and channel vocoding on alpha power [16a]: only for 16-channel vocoding, alpha power decreased 4.34% for every unit increase in SNR[16b].<br>Linear SNR term in LME regression model: interaction between vocoding and SNR on pupil size [16c]: for the 6-channel vocoding, pupil size increased 0.007 mm per unit increase in SNR[16d] and for 16-channel vocoding, pupil size decreased 0.008 mm per unit increase in SNR[16e].<br>**A cubic relationship between pupil size change and SNR was found for 6-channel vocoding**[16f], but not for 16-channel vocoding[16g].	**16a.** Slope(SE)=-4.352 (1.756) , t=-2.478 , *p*=0.014.<br>**16b.** 95%CI: -1.94% to -6.73%***.<br>**16c.** Slope(SE)=-0.016 (0.004) , t=-3.470***.<br>**16d.** 95%CI: 0.001 to 0.014 mm, *p*=0.02.<br>**16e.** 95%CI: -0.015 to -0.002 mm**.<br>**16f.** *p*=0.01.<br>**16g.** *p*=0.46.	No correlations between EEG and pupil data for both the 6-channel (mean *r*=-0.10, 95%CI: -0.35 to 0.16) and 16-channel vocoded (mean *r*=0.05, 95%CI: -0.16 to 0.26) sentences.
17	Miles et al. (2017)	N=19 NH (22-34yrs (M=27); 36.8% males).	Pupil dilation; EEG alpha power.	Speech (noise-vocoded sentences) in noise (80% and 50% intelligibility, 6-channel and 16-channel vocoding; four talker babble).<br>Actual performance levels (%) were used as they did not match the estimated intelligibility level.<br>Task: sentence repetition.	Actual performances were 64.7% (±14.7%) and 46.1% (±17.4%).<br>**Pupil size decreased by 0.05% for higher performance levels**[17a] and was 0.99% larger in the 6-channel conditions compared to the 16-channel conditions[17b].<br>No main effect of performance level[17c] or vocoding[17d] on alpha power was found. However, the LME model did not improve when adding performance level, as opposed to vocoding alone[17e]. The LME model including **only vocoding** showed that **alpha power was 29.9% larger in the 16-channel conditions** compared to the 6-channel conditions[17f].<br>The LME model including sentence recall accuracy and vocoding indicated effects of accuracy[17g] and vocoding[17h] on pupil size. No effects of either accuracy[17i] or vocoding[17j] was found on alpha power.<br>Adding SNR as a predictor in the LME model yielded no effect on pupil size[17k] or alpha power[17l].	**17a.** 95%CI: [-0.072, -.019]***.<br>**17b.** 95%CI: [0.119, 1.852], *p*=0.03.<br>**17c.** 95%CI: [-0.722, 1.001], *p*=0.75.<br>**17d.** 95%CI: [-57.310, 0.788], *p*=0.06.<br>**17e.** Model with performance level and vocoding: log likelihood -24,869.65.<br>Model with vocoding: log likelihood -24,869.79.<br>**17f.** *p*=0.03.<br>**17g.** 95%CI: [0.676, 2.549]***.<br>**17h.** 95%CI: [0.644, 2.499]***.<br>**17i.** 95%CI: [-23.206, 38.528], *p*=0.626.<br>**17j.** 95%CI: [-48.336, 12.670], *p*=0.252.<br>**17k**. 95% CI: [-0.153, 0.189], *p*=0.84.<br>**17l.** 95% CI: [-9.112, 2.191], *p*=0.23.	No correlations between pupil size and alpha power for any of the conditions (all *p*>0.05):<br>- 50% intelligibility + 6-channel vocoding: mean *r*=0.02, SD=0.14.<br>- 50% intelligibility + 16-channel vocoding: mean *r*=0.08, SD=0.20.<br>- 80% intelligibility + 6-channel vocoding: mean *r*=-0.04, SD=0.15.<br>- 80% intelligibility + 16-channel vocoding: mean *r*=-0.01, SD=0.17.<br><br>Collapsed over all conditions: mean *r*=0.01, SD=0.08, *p*>0.05.
18	Muller et al. (2019)	N=20 NH (19-35yrs (M=25); 50% males).	Peak pupil dilation;<br>EEG (neural tracking).	Speech (sentences) in noise (80% intelligibility, slow vs fast rate, three different sentence structures).<br>Task: picture matching paradigm (individual SNR at 80% determined using a sentence recognition task with sentence repetition).	Main effect of speech rate[18a] and sentence structure[18b] on SNR threshold at 80% intelligibility: SNR was higher for fast speech compared to both normal[18c] and slow speech[18d] and SNR was lower for the easier sentence structure compared to the other two structures[18e].<br>**PPD was higher for fast speech** compared to slow speech[18f], but no effect of sentence structure on PPD[18g].<br>**Neural tracking of the fast and slow rates showed different time clusters**: a negative cluster $N1_{crosscorr}$ at	**18a.** $F_{(2,36)}$=15.002***, η^2_p=0.455.<br>**18b.** $F_{(2,36)}$=6.97, *p*=0.003, η^2_p=0.279.<br>**18c and 18d.** *p*=0.001.<br>**18e.** Subject-verb-object vs object-verb-subject sentences: *p*=0.03**;**<br>Subject-verb-object vs ambiguous object-verb-subject sentences: *p*=0.02**.**	No correlation between PPDs and neural tracking (r=0.29, *p*=0.21).

					0.072–0.196 s[18h] and a positive cluster $P2_{crosscorr}$ at 0.244-0.352 s[18i]. No effect of sentence structure on neural tracking[18j].	**18f.** $F_{(1,18)}$=15.831***, η^2_p=0.468.<br>**18g.** $F_{(2,36)}$=0.22, *p*=0.08, η^2_p=0.012.<br>**18h.** 61 electrodes***.<br>**18i.** 45 electrodes***.<br>**18j.** *p*>0.025.	
19	Plain et al. (2021)	N=29 HI (47-76yrs (M=64.55); 58.6% males).	PEP;<br>HRV (HF; RMSSD);<br>HR;<br>BP.	Speech (sentences) in noise (80% and 50% intelligibility; four talker babble), with or without observers.<br>Task: sentence repetition.	Average SNR at 50% intelligibility was 5.18 (±2.09) dB and at 80% intelligibility 9.87 (±3.00) dB. Performance was lower in the 50% intelligibility condition[19a]. No main effect of social observation was found[19b].<br>A main effect of social observation was found on the cardiovascular measures[19c], but there was no main effect of intelligibility[19d].<br>Specifically, **SBP**[19e], **DPB**[19f] and **MAP**[19g] **increased when an observer was present**, but there was **no effect on PEP**[19h], **HF-HRV**[19i], **RMSSD**[19j] or **HR**[19k].	**19a.** $F_{(1,22)}$=185.54**, η^2_p=0.89.<br>**19b.** $F_{(1,22)}$=1.00, *p*=0.33, η^2_p=0.04.<br>**19c.** Wilks' lambda=0.39, $F_{(7,16)}$=3.61, p=0.02, η^2_p=0.61.<br>**19d.** Wilks' lambda=0.65, $F_{(7,16)}$=1.25, p=0.34, η^2_p=0.35.<br>**19e.** $F_{(1,22)}$= 6.70, *p*=0.04, η^2_p=0.23.<br>**19f.** $F_{(1,22)}$=10.00, *p*=0.02, η^2_p=0.31.<br>**19g.** $F_{(1,22)}$=14.08**, η^2_p=0.39.<br>**19h.** $F_{(1,22)}$=0.88, *p*=0.42, η^2_p=0.04.<br>**19i.** $F_{(1,22)}$=0.03, *p*=0.88, η^2_p=0.01.<br>**19j.** $F_{(1,22)}$=5.34, *p*=0.054, η^2_p=0.20.<br>**19k.** $F_{(1,22)}$=1.53, *p*=0.32, η^2_p=0.07.	*Per condition correlations:*<br>**Alone-difficult**: **PEP** with: SBP (r=-.40), DBP (r=-.38), and MAP (r=-.57). **HR** with: SBP (r=.40) and DBP (r=.38).<br>**Alone-easy**: **RMSSD** with: SBP (r=.37), DBP (r=.42), and MAP (r=.32). **PEP** with: HF HRV (r=-.43), SBP (r=-.48), DBP (r=-.41), and MAP (r=-.55).<br>**Observed-difficult: PEP** with: HR (r=-.42), SBP (r=-.53), DBP (r=-.59), and MAP (r=-.46). **HR** with SBP (r-.44).<br>**Observed-easy: PEP** with: HR (r=-.32), DBP (r=-.31), and MAP (r=-.48). **HR** with: SBP (r=.35), DBP (r=.60), and MAP (r=.59).<br>Aside from (expected) correlations between SBP, DBP, and MAP, all other correlations <0.3.<br>*Average correlations:*<br>**PEP** with: SBP (r=-.41), DBP (r=-.43), and MAP (r=-.52). **HR** with DBP (r=0.31). Aside from (expected) correlations between SBP, DBP, and MAP, all other correlations <0.3.
20	Richter (2016)	N=16 NH (mean age=23.88yrs; 12.5% males).	PEP;<br>BP;<br>HR.	Auditory discrimination task (discriminate between two sine waves with the same or different frequencies; easy vs difficult condition; low vs high reward).	Performance (correct discrimination) was 91.59% (±3.84) for easy-low reward, 94.53% (±2.50) for easy-high reward, 60.34% (±3.66) for difficult-low reward, and 64.47% (±3.09) for difficult-high reward.<br>Main effect of task difficulty[20a] on performance, but no main effect of reward[20b].<br>**PEP reactivity**[20c] (i.e. PEP decrease from baseline) and **HR reactivity**[20d] (i.e. HR increase from baseline)	**20a.** $F_{(1,15)}$=121.03***.<br>**20b.** $F_{(15)}$<3.22, *p*>0.09.<br>**20c.** $t_{(15)}$=4.02***.<br>**20d.** $t_{(15)}$=1.94, *p*=0.04.<br>**20e.** $ts_{(15)}$<0.95, *ps*>0.18.	

					**were higher in the high-difficulty-high-reward** condition compared to other conditions.<br>No differences for SBP and DBP[20d].		
21	Seeman and Sims (2015)	N=46 NH (18-38yrs (M=21.2); 8.7% males) participants divided over 3 experiments.	SCL;<br>HRV (SDNN);<br>HR.	<u>Experiment 1</u>: Diotic-dichotic digits task (low to high complexity: diotic single digit, dichotic single digit, dichotic double digit).<br>Baseline: short resting period<br><u>Experiment 2</u>: Speech (sentences) in noise (+15, +10, +5, 0 dB SNR; four talker babble).<br>Task: sentence repetition.<br>Baseline: short resting period	<u>Experiment 1</u>: Performance was on average 100% (±0.00) for low difficulty, 98.20% (±1.96) for medium difficulty, and 97.10% (±2.61) for high difficulty condition.<br>Main effect of task complexity on HRV reactivity[21a]: **HRV reactivity** (i.e. decrease from baseline) **increased** during dichotic single- and double-digit condition, but not for diotic digits[21b].<br>Main effect of task complexity on **HR** reactivity[21c]: **higher reactivity with increasing task complexity**.<br>**SCL reactivity did not differ** between conditions[21d]. However, a main effect of task complexity on raw SCL was found[21e]: **raw SCL was higher** for the double-digits dichotic compared to diotic digits condition[21f].<br><u>Experiment 2</u>: Performance was 98.67% (±1.17) for +15 dB SNR, 95.14% (±3.13) for +10 dB SNR, 80.00% (±18.01) for +5 dB SNR, and 17.33% (±10.16) for +0 dB SNR.<br>**Main effect of SNR on HRV reactivity**[21g]: HRV reactivity (i.e. decrease from baseline) differed between 0 and +10, 0 and +15, +5 and +10, and +5 and +15 dB SNRs[21h].<br>Main effect of SNR on HR reactivity [21i]: **HR reactivity increased** for +10 dB SNR compared to +5 dB SNR[21j].<br>**No main effect of SNR on both SCL reactivity**[22k] and **raw SCL**[22l].	**21a.** $F_{(2, 28)}$=18.54***, η^2=0.74.<br>**21b.** *p*<0.01.<br>**21c.** $F_{(2, 28)}$=4.69*, η^2=0.25.<br>**21d.** $F_{(2, 28)}$=2.30, *p*=0.12, η^2=0.15.<br>**21e.** $F_{(2, 28)}$=9.13**, η^2=0.40.<br>**21f.** *p*<0.05.<br>**21g.** $F_{(3, 42)}$=16.60***, η^2=0.54.<br>**21h.** *p*<0.01.<br>**21i.** $F_{(3, 42)}$=3.03*, η^2=0.18.<br>**21j.** *p*<0.05.<br>**21k.** $F_{(3, 42)}$=0.11, *p*=0.95, η^2=0.04.<br>**21l.** $F_{(3, 42)}$=0.52, *p*=0.67, η^2=0.04.	
22	Seifi Ala et al. (2020)	N=8 HI (mean age=70(±12) yrs; 75.0% males).	Pupil dilation;<br>EEG oscillatory power.	Speech (new clips) in noise (0 and -5 dB SNR; distractor speech and four talker babble noise).<br>Accuracy scores for comprehension questions.	Performance was higher for 0 dB SNR (76.7%) than for -5 dB SNR (61.8%)[22a].<br>**Larger MPD** for harder SNRs compared to the easier SNRs[22b].<br>No effect of SNR on the differences between time-windowed MPD (within conditions)[22c].<br>**Parietal alpha power was lower** for -5 dB SNR than for 0 dB SNR[22d].<br>Frontal theta power did not differ between conditions[22e].	**22a.** $t_{(7)}$=5.56, *p*=0.001.<br>**22b.** $F_{(1,46)}$=18.65***.<br>**22c.** $F_{(1,46)}$=2.69, *p*=0.108.<br>**22d.** $F_{(1,46)}$=4.63, *p*=0.037.<br>**22e.** $F_{(1,46)}$=0.86, *p*=0.358.	The partial correlation between the difference of MPD and alpha power with Time factor as covariance was not significant (*r*(45)=-0.168, *p*=0.258).
23	Sendesen et al. (2023)	N=15 NH with tinnitus (22-32yrs (M=24.32 ±3.28); 46.7% males);<br>N=24 NH without tinnitus (21-32yrs (M=23.48	Peak pupil dilation;<br>EEG alpha power.	Speech (noise-vocoded sentences) in noise (80% intelligibility, 16-channel vocoding; multitalker babble).<br>Task: sentence repetition.	Mean SNR of the tinnitus group was 2.34 (±.68) dB and the control group 2.43 (±.71) dB. No difference between groups[23a].<br>EEG **alpha power increase** relative to baseline was **higher for control** (M=224.13%, SD=87.46) compared to tinnitus group (M=115.64%, SD=63.13)[23b].<br>Maximum **pupil dilation** relative to baseline was	**23a.** *p*>.05.<br>**23b.** *p*=.01.<br>**23c.** *p*=.01.	

		±3.75); 45.8% males).			**higher for control** (M=11.24%, SD=3.27) compared to tinnitus group (M=8.89%, SD=3.15)[23c].		
24	Silcox and Payne (2021)	N=44 NH (18-34yrs (M=20.6); 47.7% males).	Pupil dilation; EEG N400 ERP amplitude.	Speech (sentences) in quiet and noise (power spectrum matched noise at +3 dB SNR; 3 different context conditions:<br>1) high constraint, expected final word [HighExp],<br>2) high constraint, unexpected final word [HighUnexp],<br>3) low constraint, unexpected final word [LowUnexp]).<br>Task: sentence recognition and final word recall memory tests.	Main effect of context on sentence recognition memory[24a], but not of noise[24b]. Interaction between context and noise[24c]: for LowUnexp only, recognition was better in quiet compared to noise[24d]. For final word recall, main effect of context[24e] and noise[24f]. Recall performance was better for HighExp compared to both HighUnexp[24g] and LowUnexp[24h] and better in quiet compared to noise[24i].<br>A main effect of context[24j] on N400 amplitude was found, but no main effect of noise[24k]. There was interaction between context and noise[24l]: overall, the **N400 amplitude for HighExp was lower** compared to LowUnexp [24m] and HighUnexp[24n], both in noise and in quiet. For both noise[24o] and quiet[24p], the **N400 amplitude for LowUnexp and HighUnexp did not differ**. **Only for HighUnexp, N400 amplitude was lower in noise** compared to quiet[24q], but there were no noise effects on LowUnexp [24r] or HighExp [24s].<br>Pupil sizes across the context conditions were collapsed: noise influenced the pupil response[24t], the **pupil size was larger for noise** compared to quiet[24u].	**24a.** $\chi^2_{(1)}$=93.62***.<br>**24b.** $\chi^2_{(1)}$=2.93, *p*=0.09.<br>**24c.** $\chi^2_{(1)}$=3.96*.<br>**24d.** $t_{(117)}$=2.60*.<br>**24e.** $\chi^2_{(2)}$=213.07**.<br>**24f.** $\chi^2_{(1)}$=5.60*.<br>**24g.** $t_{(195)}$=13.51**.<br>**24h.** $t_{(195)}$=19.01**.<br>**24i.** $t_{(195)}$=2.35*.<br>**24j.** $\chi^2_{(2)}$=41.43**.<br>**24k.** $\chi^2_{(1)}$=0.19, *p*=0.67.<br>**24l.** $\chi^2_{(2)}$=6.09*.<br>**24m.** Quiet: $t_{(40)}$=3.59**.<br>Noise: $t_{(40)}$=4.65**.<br>**24n.** Quiet: t(40)=6.35**.<br>Noise: $t_{(40)}$=3.55**.<br>**24o.** $t_{(40)}$=0.97, *p*=0.39.<br>**24p.** $t_{(40)}$=-1.69, *p*=0.15.<br>**24q.** $t_{(40)}$=2.21, *p*=0.05.<br>**24r.** $t_{(40)}$=-1.10, *p*=0.38.<br>**24s.** $t_{(40)}$=-0.35, *p*=0.90.<br>**24t.** $\chi^2_{(1)}$=9.59**.<br>**24u.** $t_{(43)}$=3.24**.	In noise, coupling analysis showed a relationship between HighExp N400 amplitude and pupil size (simple slope estimate=0.69, 95% CI [0.13, 1.24]) and a relationship between HighUnexp N400 amplitude and pupil size (simple slope estimate=-1.27, 95% CI [-1.27, -0.15]). No relationship between LowUnexp and pupil size (simple slope estimate=0.30, 95% CI [-0.28, 0.88]).<br>Simple slope contrasts:<br>HighExp vs HighUnexp: Dif. Est.=1.40, *z*=3.45**.<br>HighExp vs LowUnexp: Dif. Est.=0.39, *z*=0.95, *p*=0.34.<br>HighUnexp vs LowUnexp: Dif. Est.=-1.01, *z*=-2.45*.
25	Slade et al. (2021)	N=45 NH (mean age=24.87(±5.76) yrs; 42.2% males).	PEP;<br>RSA;<br>HR;<br>BP.	Speech (stories) in noise (+24, +2, -4, -16 dB SNR; white noise).<br>Accuracy scores for comprehension questions.	The linear planned contrasts were significant for performance[25a]: average performance for the +24 dB condition was 96.7% (±1.0%), for +2 dB 78.7% (±1.8%), for -4 dB 76.2% (±2.4%), and for -16 dB 27.3% (±2.8%).<br>A **quadratic relationship between SNR changes and PEP reactivity** was found[25b].<br>**No significant planned contrast for RSA**[25c] and **respiration-centered RSA**[25d] (the RSA based on the individual respiration frequency).<br>The planned contrasts testing a **sawtooth model for the effect of SNR on HR**[25e]**, SBP**[25f], and **DBP**[25g] **were not significant**.	**25a.** $t_{(132)}$=22.60***, $r_{contrast}$=0.96.<br>**25b.** $t_{(132)}$=2.05, *p*=0.02, $r_{contrast}$=0.30.<br>**25c.** $t_{(132)}$=1.58, *p*=0.06, $r_{contrast}$=0.24.<br>**25d.** $t_{(102)}$=1.18, *p*=0.12, $r_{contrast}$=0.20.<br>**25e.** $t_{(132)}$=0.29, *p*=0.39, $r_{contrast}$=0.04.<br>**25f.** $t_{(132)}$=0.62, *p*=0.27, $r_{contrast}$=0.09.<br>**25g.** $t_{(132)}$=0.91, *p*=0.18, $r_{contrast}$=0.14.	
26	Zekveld et al. (2014)	N=17 NH (19-33yrs (M=24.2); 50% males).	Peak pupil dilation;<br>fMRI BOLD response.	Speech (sentences) in quiet and in noise (84% and 50% intelligibility; 3 degradation types: fluctuating noise, interfering single-talker speech, noise-vocoding procedure).	For 50% intelligibility, the mean SNR was -3.91(±1.70) dB for single-talker and -3.85(±1.37) dB for fluctuating noise. For 84% intelligibility, mean SNR was 0.41 (±2.43) dB for single-talker and 0.22 (±1.49) dB for fluctuating noise. Main effect of intelligibility[26a], but not masker type[26b] on SNR.<br>For noise vocoded speech, the average number of	**26a.** $F_{(1,16)}$=252.4***.<br>**26b.** *p*=0.86.<br>**26c.** $t_{(16)}$=-6.80***.<br>**26d.** $F_{(1,16)}$=47.4***.<br>**26e.** $F_{(1,16)}$=10.7, *p*=0.005.<br>**26f.** $F_{(2,32)}$=8.56***.<br>**26g.** single talker vs fluctuating noise: $F_{(1,16)}$=6.8, *p*=0.02;	Positive association between BOLD and PPD in bilateral STG, bilateral ventral and dorsal cingulate gyrus, bilateral superior frontal gyrus, bilateral precentral gyrus, bilateral frontal operculum, right putamen

				2 test sessions: session 1 with pupillometry and session 2 with fMRI and pupillometry.<br>Task: sentence repetition.	bands for 50% (6.9 (±.81)) differed from those for 84% (9.8 (±1.6)) [26c].<br>Main effect of test session[26d], intelligibility level[26e] and degradation type[26f] on PPD. All three degradation types differed for PPD[26g]: **PPD for single talker > fluctuating noise > noise-vocoded speech**. **PPD was higher for 50%** compared to 84% intelligibility and PPD was higher during session 1 compared to session 2.<br>Whole brain activation: no main effect of intelligibility[26h], but **main effect of degradation type in the left precentral gyrus**[26i] and in **bilateral STG**[26j]. **Bilateral STG activity** was sensitive to degradation types[26k]: **activation during single-talker > fluctuating noise > noise-vocoded speech**.	single talker vs noise-vocoding: $F_{(1,16)}$=22.7, *p*=0.001;<br>fluctuating noise vs noise-vocoding: $F_{(1,16)}$=8.43, *p*=0.01.<br>**26h.** FWE corrected *p*=0.19.<br>**26i.** FWE corrected *p*=0.047.<br>**26j.** FWE corrected***.<br>**26k.** FWE corrected*.	and right inferior frontal gyrus (FWE corrected *p* < 0.05).
27	Zekveld et al. (2019)	4 groups:<br>1) N=17 NH control group (mean age=52(±12) yrs; 17.6% males);<br><br>2) N=17 NH feedback group (mean age=52(±10) yrs; 41.2% males);<br><br>3) N=15 HI control group (mean age=49(±13) yrs; 53.3% males);<br><br>4) N=14 HI feedback group (mean age=55(±13) yrs; 50.0% males).	Peak pupil dilation;<br>Saliva cortisol;<br>Saliva alpha-amylase.	Speech (sentences) in noise (50% and 71% intelligibility; interfering speech). With or without feedback (between subjects analysis).<br>Task: sentence repetition.<br><br>Cortisol and sAA levels were measured before testing (baseline), during the test, and after testing (post-session).	Main effect of intelligibility level[27a] and hearing status[27b] on SRT (dB SNR) and an interaction effect between feedback status and intelligibility level[27c]. No main effect of feedback status.<br>Main effect of intelligibility level[27d] and feedback status[27e] on PPD: **PPD was smaller for 71% intelligibility** compared to 50% and **larger for feedback** compared to no feedback group.<br>No **main effect of hearing status on PPD** [27f].<br>M**ain effect of intelligibility on MPD**[27g], but no main effects of feedback[27h] and hearing status[27i].<br>Main effect of measurement time on (log_{10} transformed) sAA activity[27j], but no main effects of feedback[27k] and hearing status[27l]. **sAA differed between baseline and during testing**[27m], and between baseline and post-session[27n], but not during and after testing[27o].<br>Main effect of measurement time on (log_{10} transformed) cortisol levels[27p]. Age interacted with measurement time[27q]: **cortisol levels decreased less for older participants**. Main effect of gender[27r]: females had overall higher cortisol levels[27s]. **Cortisol levels differed between baseline and during testing**[27t], and between baseline and post-session[27u], but not between during and after testing. No main effects of feedback[27v] and hearing status[27w] on cortisol levels were found.	**27a.** $F_{(1, 59)}$=136.8***, η^2_p=0.70.<br>**27b.** $F_{(1, 59)}$=77.5***, η^2_p =0.57.<br>**27c.** $F_{(1, 59)}$=5.4*, η^2_p =0.08.<br>**27d.** $F_{(1, 59)}$=5.22*, η^2_p =0.08.<br>**27e.** $F_{(1, 59)}$=4.06*, η^2_p =0.06.<br>**27f.** η^2_p =0.001.<br>**27g.** $F_{(1, 59)}$=9.30**, η^2_p =0.14.<br>**27h.** η^2_p =0.035.<br>**27i.** η^2_p =0.012.<br>**27j.** $F_{(2, 112)}$=31.2**, η^2_p =0.36.<br>**27k.** η^2_p =0.005.<br>**27l.** η^2_p =0.005.<br>**27m.** $t_{(59)}$=-5.7**.<br>**27n.** $t_{(59)}$=-6.8**.<br>**27o.** *p*=0.15.<br>**27p.** $F_{(2, 114)}$=7.96**, η^2_p =0.12.<br>**27q.** $F_{(2, 114)}$=3.8 η^2_p =0.06.<br>**27r.** $F_{(1, 57)}$=47.1**, η^2_p =0.45.<br>**27s.** Baseline: t(61)=4.27***;<br>During testing: $t_{(61)}$=6.54***;<br>Post-session: $t_{(61)}$=6.67***.<br>**27t.** $t_{(62)}$=4.66**.<br>**27u.** $t_{(62)}$=3.46**.<br>**27v.** η^2_p =0.033.<br>**27w** η^2_p =0.001.	None of the correlations between PPD and cortisol level (r=-0.06 - 0.13, *p*>0.05) and between PPD and sAA activity (r=-0.25 - -0.11, *p*>0.05) were significant, except for a weak correlation between PPD during the 71% intelligibility condition and baseline sAA activity (r=-0.26*).

28	Zhou et al. (2022)	N=28 NH (18-27yrs (M=21.6); 39.3% males).	Peak pupil dilation;<br>fNIRS cerebral oxygenation.	Sentences in quiet (4 conditions: shuffled [S], vocoded [V], shuffled-vocoded [SV], vocoded-interrupted[VI]).<br><br>Experiment 1 (pupillometry): Task: sentence repetition.<br><br>Experiment 2 (fNIRS): Task: sentence recognition (y/n).	Experiment 1: Performance differed across conditions[28a]. Repetition scores were higher for S > V > SV > VI[28b].<br>PPD differed between conditions[28c]: **PPD for S was lower** compared to SV[28d] and VI[28e], and **PPD for V was lower** compared to SV[28f] and VI[28g].<br>Experiment 2: Mean accuracy was above chance level of 50% correct in all conditions[28h].<br>Cerebral oxygenation (ΔHbC amplitude) in the **left LFC** differed between conditions[28i]: **ΔHbC amplitude was lower for VI** compared to S[28j], V[28k], and SV[28l].<br>Efect of condition in the **left AC**[28m]: **ΔHbC amplitude was lower for VI** compared to S[28n].	**28a.** $F_{(3, 81)}$=288.08***.<br>**28b.** *p*<0.001.<br>**28c.** $F_{(3, 81)}$=34.49***.<br>**28d.** $t_{(81)}$=-5.52***.<br>**28e.** $t_{(81)}$=-654***.<br>**28f.** $t_{(81)}$=-7.22***.<br>**28g.** $t_{(81)}$=-8.24***.<br>**28h.** *p*<0.001.<br>**28i.** $F_{(3, 81)}$=5.18, *p*=0.003.<br>**28j.** $t_{(81)}$=3.45, *p*=0.005.<br>**28k.** $t_{(81)}$=2.78, *p*=0.034.<br>**28l.** $t_{(81)}$=3.28, *p*=0.008.<br>**28m.** $F_{(3, 81)}$=2.96, *p*=0.037.<br>**28n.** $t_{(81)}$=2.84, *p*=0.029.	PPD did not correlate with ΔHbC amplitudes the left LFC (r=-0.05, *p*=0.681) or left AC (r=-0.10, *p*=0.386).

*p<.05; **p<.01; ***p<.001. Values stated as presented in the articles.
AC = auditory cortex; BOLD = blood oxygenation level dependent; BP = blood pressure; BVPA = blood volume pulse amplitude; CgA = Chromogranin A; dB = decibel; DBP = diastolic blood pressure; EEG = electroencephalography; EMG = electromyography; ERP = event-related potentials; ERSP = event-related spectral perturbation; FA = factor analysis; fMRI = functional magnetic resonance imaging; fNIRS = functional near-infrared spectroscopy; HF = high frequency; HI = hearing impaired; HP = heart period; HR = heart rate; HRV = heart rate variability; LF = low frequency; LFC = lateral frontal cortex; LME = linear mixed model; MPD = mean pupil dilation; NH = normal hearing; PCA = principal components analysis; PEP = pre-ejection period; PPD = peak pupil dilation; RMSSD = root mean square of successive differences; RSA = respiratory sinus arrhythmia; sAA = saliva alpha-amylase; SBP = systolic blood pressure; SCL = skin conductance level; SCR = skin conductance response; SDNN = standard deviation of all NN intervals; SNR = speech-to-noise ratio; TRF = temporal response functions; WM = working memory.

References

Alhanbali, S., Dawes, P., Millman, R. E., & Munro, K. J. (2019). Measures of Listening Effort Are Multidimensional. *Ear Hear*, *40*(5), 1084-1097. https://doi.org/10.1097/AUD.0000000000000697

Corcoran, A. W., Perera, R., Koroma, M., Kouider, S., Hohwy, J., & Andrillon, T. (2022). Expectations boost the reconstruction of auditory features from electrophysiological responses to noisy speech. *Cereb Cortex*. https://doi.org/10.1093/cercor/bhac094

Cvijanović, N., Kechichian, P., Janse, K., & Kohlrausch, A. (2017). Effects of noise on arousal in a speech communication setting. *Speech Communication*, *88*, 127-136. https://doi.org/10.1016/j.specom.2017.02.001

Fiedler, L., Seifi Ala, T., Graversen, C., Alickovic, E., Lunner, T., & Wendt, D. (2021). Hearing Aid Noise Reduction Lowers the Sustained Listening Effort During Continuous Speech in Noise-A Combined Pupillometry and EEG Study. *Ear Hear*, *42*(6), 1590-1601. https://doi.org/10.1097/AUD.0000000000001050

Francis, A. L., Bent, T., Schumaker, J., Love, J., & Silbert, N. (2021). Listener characteristics differentially affect self-reported and physiological measures of effort associated with two challenging listening conditions. *Atten Percept Psychophys*, *83*(4), 1818-1841. https://doi.org/10.3758/s13414-020-02195-9

Francis, A. L., MacPherson, M. K., Chandrasekaran, B., & Alvar, A. M. (2016). Autonomic Nervous System Responses During Perception of Masked Speech may Reflect Constructs other than Subjective Listening Effort. *Front Psychol*, *7*, 263. https://doi.org/10.3389/fpsyg.2016.00263

Giuliani, N. P., Brown, C. J., & Wu, Y. H. (2020). Comparisons of the Sensitivity and Reliability of Multiple Measures of Listening Effort. *Ear Hear*, *42*(2), 465-474. https://doi.org/10.1097/AUD.0000000000000950

Haro, S., Rao, H. M., Quatieri, T. F., & Smalt, C. J. (2022). EEG alpha and pupil diameter reflect endogenous auditory attention switching and listening effort. *Eur J Neurosci*, *55*(5), 1262-1277. https://doi.org/10.1111/ejn.15616

Hjortkjaer, J., Marcher-Rorsted, J., Fuglsang, S. A., & Dau, T. (2020). Cortical oscillations and entrainment in speech processing during working memory load. *Eur J Neurosci*, *51*(5), 1279-1289. https://doi.org/10.1111/ejn.13855

Kramer, S. E., Teunissen, C. E., & Zekveld, A. A. (2016). Cortisol, Chromogranin A, and Pupillary Responses Evoked by Speech Recognition Tasks in Normally Hearing and Hard-of-Hearing Listeners: A Pilot Study. *Ear Hear*, *37 Suppl 1*, 126S-135S. https://doi.org/10.1097/AUD.0000000000000311

Lim, S. J., Carter, Y. D., Njoroge, J. M., Shinn-Cunningham, B. G., & Perrachione, T. K. (2021). Talker discontinuity disrupts attention to speech: Evidence from EEG and pupillometry. *Brain Lang*, *221*, 104996. https://doi.org/10.1016/j.bandl.2021.104996

Mackersie, C. L., & Calderon-Moultrie, N. (2016). Autonomic Nervous System Reactivity During Speech Repetition Tasks: Heart Rate Variability and Skin Conductance. *Ear Hear*, *37 Suppl 1*, 118S-125S. https://doi.org/10.1097/AUD.0000000000000305

Mackersie, C. L., & Cones, H. (2011). Subjective and psychophysiological indexes of listening effort in a competing-talker task. *J Am Acad Audiol*, *22*(2), 113-122. https://doi.org/10.3766/jaaa.22.2.6

Mackersie, C. L., & Kearney, L. (2017). Autonomic Nervous System Responses to Hearing-Related Demand and Evaluative Threat. *Am J Audiol*, *26*(3S), 373-377. https://doi.org/10.1044/2017_AJA-16-0133

Mackersie, C. L., MacPhee, I. X., & Heldt, E. W. (2015). Effects of hearing loss on heart rate variability and skin conductance measured during sentence recognition in noise. *Ear Hear*, *36*(1), 145-154. https://doi.org/10.1097/AUD.0000000000000091

McMahon, C. M., Boisvert, I., de Lissa, P., Granger, L., Ibrahim, R., Lo, C. Y., Miles, K., & Graham, P. L. (2016). Monitoring Alpha Oscillations and Pupil Dilation across a Performance-Intensity Function. *Front Psychol*, *7*, 745. https://doi.org/10.3389/fpsyg.2016.00745

Miles, K., McMahon, C., Boisvert, I., Ibrahim, R., de Lissa, P., Graham, P., & Lyxell, B. (2017). Objective Assessment of Listening Effort: Coregistration of Pupillometry and EEG. *Trends Hear*, *21*, 2331216517706396. https://doi.org/10.1177/2331216517706396

Muller, J. A., Wendt, D., Kollmeier, B., Debener, S., & Brand, T. (2019). Effect of Speech Rate on Neural Tracking of Speech. *Front Psychol*, *10*, 449. https://doi.org/10.3389/fpsyg.2019.00449

Plain, B., Pielage, H., Richter, M., Bhuiyan, T. A., Lunner, T., Kramer, S. E., & Zekveld, A. A. (2021). Social observation increases the cardiovascular response of hearing-impaired listeners during a speech reception task. *Hear Res*, *410*, 108334. https://doi.org/10.1016/j.heares.2021.108334

Richter, M. (2016). The Moderating Effect of Success Importance on the Relationship Between Listening Demand and Listening Effort. *Ear Hear*, *37 Suppl 1*, 111S-117S. https://doi.org/10.1097/AUD.0000000000000295

Seeman, S., & Sims, R. (2015). Comparison of Psychophysiological and Dual-Task Measures of Listening Effort. *J Speech Lang Hear Res*, *58*(6), 1781-1792. https://doi.org/10.1044/2015_JSLHR-H-14-0180

Seifi Ala, T., Graversen, C., Wendt, D., Alickovic, E., Whitmer, W. M., & Lunner, T. (2020). An exploratory Study of EEG Alpha Oscillation and Pupil Dilation in Hearing-Aid Users During Effortful listening to Continuous Speech. *PLoS One*, *15*(7), e0235782. https://doi.org/10.1371/journal.pone.0235782

Sendesen, E., Kilic, S., Erbil, N., Aydin, O., & Turkyilmaz, D. (2023). An Exploratory Study of the Effect of Tinnitus on Listening Effort Using EEG and Pupillometry. *Otolaryngol Head Neck Surg*. https://doi.org/10.1002/ohn.367

Silcox, J. W., & Payne, B. R. (2021). The costs (and benefits) of effortful listening on context processing: A simultaneous electrophysiology, pupillometry, and behavioral study. *Cortex*, *142*, 296-316. https://doi.org/10.1016/j.cortex.2021.06.007

Slade, K., Kramer, S. E., Fairclough, S., & Richter, M. (2021). Effortful listening: Sympathetic activity varies as a function of listening demand but parasympathetic activity does not. *Hear Res*, *410*, 108348. https://doi.org/10.1016/j.heares.2021.108348

Zekveld, A. A., Heslenfeld, D. J., Johnsrude, I. S., Versfeld, N. J., & Kramer, S. E. (2014). The eye as a window to the listening brain: neural correlates of pupil size as a measure of cognitive listening load. *Neuroimage*, *101*, 76-86. https://doi.org/10.1016/j.neuroimage.2014.06.069

Zekveld, A. A., van Scheepen, J. A. M., Versfeld, N. J., Veerman, E. C. I., & Kramer, S. E. (2019). Please try harder! The influence of hearing status and evaluative feedback during listening on the pupil dilation response, saliva-cortisol and saliva alpha-amylase levels. *Hear Res*, *381*, 107768. https://doi.org/10.1016/j.heares.2019.07.005

Zhou, X., Burg, E., Kan, A., & Litovsky, R. Y. (2022). Investigating effortful speech perception using fNIRS and pupillometry measures. *Current Research in Neurobiology*, *3*. https://doi.org/10.1016/j.crneur.2022.100052
